# Supplementary material for: Effectiveness of physical and mental health interventions for young people with heart conditions: protocol for a systematic review and meta-analysis
Source: BMJ Open. 2023 Feb 6;13(2):e067342. doi: 10.1136/bmjopen-2022-067342 (PMC9906184; doi:10.1136/bmjopen-2022-067342)
Supplement: Supplementary data [file bmjopen-2022-067342supp002.pdf]

Appendix 2

Search Strategy (PsychInfo)

1. "psycho education" or "psychosocial" or "psychosocial*" or "Psycho* adj intervent* or program* or strategy* or support or treat* or therap* or counsel* or manage*"

2. "quality of life"

3. "exercise" or "sports" or "aerobic exercise" or "physical activity" or "cardiac rehabilitation" or "training" or "physical exercise" or "recreation" or "aerobic training" or "aerobic intervention" or "exercise prescription" or "activity" or "exercise performance" or "exercise rehab*" or "exercise programme" or "aerobic exercise training" or "physical training" or "High-Intensity Interval Training" or "HIIT" or "rehab" or "strength" or "endurance" or "resistance"

4. “congenital heart disease” OR “congenital heart diseases” OR “congenital heart defect” OR “congenital heart defects” OR “heart disease” OR “cardiomyopathy” OR “arrythmia” OR “cardiac defect”

5. “children” OR “adolescent” OR “adolescents” OR “young adult” OR “paedeatric” OR “youth”

6. 1 OR 2 OR 3

7. 4 AND 5

8. 6 AND 7
LIMIT: 1980 – Current

Search Strategy (MEDLINE)

1. "psycho education".mp. [mp=title, book title, abstract, original title, name of substance word, subject heading word, floating sub-heading word, keyword heading word, organism supplementary concept word, protocol supplementary concept word, rare disease supplementary concept word, unique identifier, synonyms]
2. "psychosocial".ab,ti.
3. "psycho* intervent*".ab,ti.
4. "psycho* program*".ab,ti.
5. "psycho* strategy ".ab,ti.
6. "psycho* support ".ab,ti.
7. "psycho* treat* ".ab,ti.
8. "psycho* therap* ".ab,ti.
9. "psycho* counsel* ".ab,ti.
10. "psycho* manage* ".ab,ti.
11. "quality of life".ab,ti.
12. "exercise".ab,ti.
13. "sports".ab,ti.
14. "aerobic exercise".ab,ti.
15. "physical activity".ab,ti.
16. "cardiac rehabilitation".ab,ti.
17. "physical exercise".ab,ti.
18. "recreation".ab,ti.
19. "aerobic training".ab,ti.
20. "aerobic intervention".ab,ti.
21. "exercise prescription".ab,ti.
22. "exercise performance".ab,ti.
23. "exercise rehab* ".ab,ti.
24. "exercise programme".ab,ti.
25. "aerobic exercise training".ab,ti.
26. "physical training".ab,ti.
27. "high-intensity interval training".ab,ti.
28. "HIIT".ab,ti.

29. "rehab".ab,ti.
30. "strength".ab,ti.
31. "endurance".ab,ti.
32. "congenital heart disease".ab,ti.
33. "congenital heart diseases".ab,ti.
34. "congenital heart defects".ab,ti.
35. "congenital heart defect".ab,ti.
36. "heart disease".ab,ti.
37. "cardiomyopathy".ab,ti.
38. "arrythmia".ab,ti.
39. "cardiac defect".ab,ti.
40. "children".ab,ti.
41. "adolescen* ".ab,ti.
42. "young adult".ab,ti.
43. "paedeatric".ab,ti.
44. "youth".ab,ti.
45. 1 or 2 or 3 or 4 or 5 or 6 or 7 or 8 or 9 or 10
46. 12 or 13 or 14 or 15 or 16 or 17 or 18 or 19 or 20 or 21 or 22 or 23 or 24 or 25 or 26 or 27 or 28 or 29 or 30 or 31
47. 32 or 33 or 34 or 35 or 36 or 37 or 38 or 39
48. 40 or 41 or 42 or 43 or 44
49. 11 or 45 or 46
50. 47 and 48
51. 49 and 50
52. limit 51 to (yr="1980 -Current" and (english or italian))
53. limit 52 to ("all child (0 to 18 years)" or "newborn infant (birth to 1 month)" or "infant (1 to 23 months)" or "preschool child (2 to 5 years)" or "child (6 to 12 years)" or "adolescent (13 to 18 years)")

Search Strategy (EMBASE)

1. "psycho education".mp. [mp=title, abstract, heading word, table of contents, key concepts, original title, tests & measures, mesh word]
2. "psychosocial".ab,ti.
3. "psycho* intervent*".ab,ti.
4. "psycho* program*".ab,ti.
5. "psycho* strategy ".ab,ti.
6. "psycho* support ".ab,ti.
7. "psycho* treat* ".ab,ti.
8. "psycho* therap* ".ab,ti.
9. "psycho* counsel* ".ab,ti.
10. "psycho* manage* ".ab,ti.
11. "quality of life".ab,ti.
12. "exercise".ab,ti.
13. "sports".ab,ti.
14. "aerobic exercise".ab,ti.
15. "physical activity".ab,ti.
16. "cardiac rehabilitation".ab,ti.
17. "physical exercise".ab,ti.
18. "recreation".ab,ti.
19. "aerobic training".ab,ti.
20. "aerobic intervention".ab,ti.
21. "exercise prescription".ab,ti.
22. "exercise performance".ab,ti.
23. "exercise rehab* ".ab,ti.
24. "exercise programme".ab,ti.
25. "aerobic exercise training".ab,ti.
26. "physical training".ab,ti.
27. "high-intensity interval training".ab,ti.
28. "HIIT".ab,ti.
29. "rehab".ab,ti.
30. "strength".ab,ti.
31. "endurance".ab,ti.

32. "congenital heart disease".ab,ti.

33. "congenital heart diseases".ab,ti.

34. "congenital heart defect".ab,ti.

35. "congenital heart defects".ab,ti.

36. "cardiomyopathy".ab,ti.

37. "arrythmia".ab,ti.

38. "cardiac defect".ab,ti.

39. "children".ab,ti.

40. "adolescen* ".ab,ti.

41. "young adult".ab,ti.

42. "youth".ab,ti.

43. "paedeatric".ab,ti.

44. 1 or 2 or 3 or 4 or 5 or 6 or 7 or 8 or 9 or 10

45. 12 or 13 or 14 or 15 or 16 or 17 or 18 or 19 or 20 or 21 or 22 or 23 or 24 or 25 or 26 or 27 or 28 or 29 or 30 or 31

46. 32 or 33 or 34 or 35 or 36 or 37 or 38

47. 39 or 40 or 41 or 42 or 43

48. 11 or 44 or 45

49. 44 or 45

50. 46 and 47

51. 48 and 50

52. 49 and 50

53. limit 52 to (english language or italian)

54. limit 53 to (child <unspecified age> or preschool child <1 to 6 years> or school child <7 to 12 years> or adolescent <13 to 17 years>)

Search Strategy (Web of Science, including Science Citation Index)

1: TS= ("psycho education" or "psychosocial" or "psychosocial*" or "Psycho* adj intervent* or program* or strategy* or support or treat* or therap* or counsel* or manage*")
Results: 142860

2: TS=("quality of life")
Results: 493264

3: TS=("exercise" or "sports" or "aerobic exercise" or "physical activity" or "cardiac rehabilitation" or "exercise training" or "physical exercise" or "recreation" or "aerobic training" or "aerobic intervention" or "exercise prescription" or "exercise performance" or "exercise rehab*" or "exercise programme" or "aerobic exercise training" or "physical training" or "High-Intensity Interval Training" or "HIIT" or "rehab" or "strength" or "endurance")

4: TS=("congenital heart disease" OR "congenital heart diseases" OR "congenital heart defect" OR "congenital heart defects" OR "heart disease" OR "cardiomyopathy" OR "arrythmia" OR "cardiac defect")
Editions: WOS.SCI

5: TS=("children" OR "adolescent" OR "adolescents" OR "young adult" OR "paedeatric" OR "youth").
Editions: WOS.SCI

6: #1 OR #3 Editions: WOS.SCI

7: #4 AND #5 Editions: WOS.SCI

8: #6 AND #7 Editions: WOS.SCI

9: #6 AND #7 and 1921 or 1923 or 1927 or 1965 or 1969 or 1970 or 1972 or 1974 or 1975 or 1977 or 1979 (Exclude – Publication Years) Editions: WOS.SCI

10: #6 AND #7 and 1921 or 1923 or 1927 or 1965 or 1969 or 1970 or 1972 or 1974 or 1975 or 1977 or 1979 (Exclude – Publication Years) and English or Italian (Languages) Editions: WOS.SCI

Search Strategy (Cochrane Central Register of Controlled Trials (CENTRAL))

#1 ("psycho education" or "psychosocial" or "psychosocial*" or "Psycho* adj intervent* or program* or strategy* or support or treat* or therap* or counsel* or manage*"):ti,ab,kw (Word variations have been searched)

#2 quality of life

#3 ("exercise" or "sports" or "aerobic exercise" or "physical activity" or "cardiac rehabilitation" or "training" or "physical exercise" or "recreation" or "aerobic training" or "aerobic intervention" or "exercise prescription" or "activity" or "exercise performance" or "exercise rehab*" or "exercise programme" or "aerobic exercise training" or "physical training" or "High-Intensity Interval Training" or "HIIT" or "rehab" or "strength" or "endurance" or "resistance"):ti,ab,kw

#4 “congenital heart disease” OR “congenital heart diseases” OR “congenital heart defect” OR “congenital heart defects” OR “heart disease” OR “cardiomyopathy” OR “arrythmia” OR “cardiac defect”

#5 “children” OR adolescen* OR “young adult” OR “paedeatric” OR “youth”

#6 #1 OR #2 OR #3

#7 #4 and #5

#8 #6 and #7 with Publication Year from 1980 to 2022, in Trials

Search strategy - DARE (via the Cochrane Library)

#1 ("psycho education" or "psychosocial" or "psychosocial*" or "Psycho* adj intervent* or program* or strategy* or support or treat* or therap* or counsel* or manage*"):ti,ab,kw (Word variations have been searched)

#2 quality of life

#3 ("exercise" or "sports" or "aerobic exercise" or "physical activity" or "cardiac rehabilitation" or "training" or "physical exercise" or "recreation" or "aerobic training" or "aerobic intervention" or "exercise prescription" or "activity" or "exercise performance" or "exercise rehab*" or "exercise programme" or "aerobic exercise training" or "physical training" or "High-Intensity Interval Training" or "HIIT" or "rehab" or "strength" or "endurance" or "resistance"):ti,ab,kw

#4 “congenital heart disease” OR “congenital heart diseases” OR “congenital heart defect” OR “congenital heart defects” OR “heart disease” OR “cardiomyopathy” OR “arrythmia” OR “cardiac defect”

#5 “children” OR adolescen* OR “young adult” OR “paedeatric” OR “youth”
#6 #1 OR #2 OR #3
#7 #4 and #5
#8 #6 and #7 with Publication Year from 1980 to 2022, in Cochrane Reviews
