# Supplementary material for: Effectiveness of physical and mental health interventions for young people with heart conditions: protocol for a systematic review and meta-analysis
Source: BMJ Open. 2023 Feb 6;13(2):e067342. doi: 10.1136/bmjopen-2022-067342 (PMC9906184; doi:10.1136/bmjopen-2022-067342)
Supplement: Author's manuscript [file bmjopen-2022-067342.draft_revisions.pdf]

BMJ Open is committed to open peer review. As part of this commitment we make the peer review history of every article we publish publicly available.

When an article is published we post the peer reviewers' comments and the authors' responses online. We also post the versions of the paper that were used during peer review. These are the versions that the peer review comments apply to.

The versions of the paper that follow are the versions that were submitted during the peer review process. They are not the versions of record or the final published versions. They should not be cited or distributed as the published version of this manuscript.

BMJ Open is an open access journal and the full, final, typeset and author-corrected version of record of the manuscript is available on our site with no access controls, subscription charges or pay-per-view fees (http://bmjopen.bmj.com).

If you have any questions on BMJ Open's open peer review process please email info.bmjopen@bmj.com

BMJ Open

Protocol for a systematic review and meta-analysis of the Effectiveness of Physical and Mental Health Interventions for Young People with Heart Conditions.

Journal:	*BMJ Open*
Manuscript ID	bmjopen-2022-067342
Article Type:	Protocol
Date Submitted by the Author:	09-Aug-2022
Complete List of Authors:	Capobianco, Lora; Greater Manchester Mental Health NHS Foundation Trust, Research and Innovation; The University of Manchester<br>Adewusi, Joy; Greater Manchester Mental Health NHS Foundation Trust, Research and Innovation<br>Cooper, Beth; Greater Manchester Mental Health NHS Foundation Trust, Research and Innovation<br>Belcher, Andrew; Greater Manchester Mental Health NHS Foundation Trust, Research and Innovation<br>Wells, Adrian; The University of Manchester, Department of Clinical Psychology
Keywords:	MENTAL HEALTH, CARDIOLOGY, REHABILITATION MEDICINE, Congenital heart disease < CARDIOLOGY, Child & adolescent psychiatry < PSYCHIATRY

SCHOLARONE™
Manuscripts

Protocol for a systematic review and meta-analysis of the Effectiveness of Physical and Mental Health Interventions for Young People with Heart Conditions.

Lora Capobianco [1,2]

Joy Adewusi [1]

Beth Cooper [1]

Andrew Belcher [1]

Adrian Wells [2]

[1] Research and Innovation, Greater Manchester Mental Health NHS Foundation Trust, Manchester, UK

[2] School of Psychological Sciences, Faculty of Biology, Medicine and Health, The University of Manchester

Corresponding Author: Lora Capobianco, lora.capobianco@gmmh.nhs.uk

Word count: 2793, Figures: 0; Tables: 0

For peer review only

Abstract

Introduction: Cardiovascular disease is amongst the most common of non-communicable diseases, affecting 13.9 million children and young people (CYP) globally. Survival rates for CYP with heart conditions are rising, however support for adjusting to life with a heart condition is lacking, as such it is unsurprising that 1 in 3 suffer from anxiety, depression, or adjustment disorder. The proposed review aims to identify and assess the effectiveness of physical and mental health interventions across physical and mental health outcomes in young people with cardiac conditions using narrative synthesis and meta-analysis if appropriate.

Methods and analysis: Embase, Medline, PubMed, PsycINFO, Cochrane Databases, Web of Science and reference lists of relevant publications will be searched from inception to June 2022 for articles published in English or Italian. Screening, data extraction, intervention coding and risk of bias will be performed by two independent reviewers using an extraction checklist. Intervention content and features will be identified and reported using the Template for Intervention Description and Replication (TIDieR) checklist. A narrative review of the included studies will be conducted. If possible and appropriate, a random-effects model meta-analysis will be conducted to calculate the pooled within-group and between-group effect sizes for the primary outcome measures. If sufficient data is available, a sub-group meta-analysis will investigate whether specific intervention types are associated with intervention effectiveness.

Ethics and Dissemination: This systematic review does not directly involve the use of human beings therefore there is no requirement for ethical approval. Findings will be disseminated through peer-reviewed publication and in various media, such as conferences, congresses or symposia.

PROSPERO registration number: CRD42022330582

For peer review only

Strengths and limitations of this study:

- The review addresses a gap in the literature by being the first to explore the effectiveness of mental and physical intervention on mental and physical outcomes in young people with a heart condition overcoming a limitation of previous reviews.
- The review is not limited to randomised trials but will incorporate a range of study designs including benchmarking studies, non-randomised interventional studies, pilot studies, uncontrolled trials, case series studies, and randomised trials.
- This protocol reduces the possibility of duplication, gives transparency to the methods and processes the review team will use, reduces the risk of biases and allows for peer review.
- The study protocol is informed by the Cochrane Collaboration systematic review methods and adheres to the Preferred Reporting Items for Systematic Reviews and Meta-Analyses Protocols.

For peer review only

Introduction

Childhood heart conditions such as congenital heart disease, cardiomyopathy, and arrythmias, are amongst the most common childhood non communicable diseases, impacting 13.8 million children and young people (CYP) under the age of 20 globally [1]. Survivors of childhood heart conditions face challenges and comorbidities which impact their ability to function [2], future employment, and progression into independent adulthood [3], [4]. CYP with heart conditions are also less likely to engage in physical activity in comparison with healthy controls [5], which increases their risk for atherosclerosis, cardiovascular disease, obesity, and diabetes [6].

Recommendations from the American Heart Association and European Association of Cardiovascular Prevention and Rehabilitation, [7] have noted the importance of CYP engaging in physical activity, exercise and maintaining a healthy lifestyle. Regular moderate to vigorous physical activity in CYP has been shown to improve both physical and mental health [8]. This is of particular importance as CYP with heart conditions have a reduced quality of life [9]–[11] and poorer psychological functioning, with 30% suffering from anxiety or depression [10], [12]–[15] and 41% experience psychological maladjustment [10].

Interventions to improve outcomes in CYP with CHD have predominantly focused on improving exercise capacity or engagement in physical activity [16]. Van Deutekom and Lewandowski (2021) [16] conducted a narrative review of physical activity modification in CYP with CHD. Eight interventions were included that aimed to improve physical activity. The authors noted the heterogeneity in the interventions offered, with four of the trials reporting a significant increase in physical activity levels following the intervention. These results are in line with previous systematic reviews which have also noted that physical activity and exercise training programmes significantly improve physical health outcomes (i.e. peak VO_2, activity levels and muscle strength) [17].

For peer review only

While systematic reviews have noted that exercise and physical activity training improve physical health outcomes, only one review [18] has commented on improvements in quality-of-life and one review has been conducted to evaluate mental health interventions for CYP with CHD [19]. Tesson *et al.* (2019) [19] conducted a systematic review of psychological interventions for childhood-onset heart disease and found only two studies focused on interventions for CYP [20], [21]. The results were disappointing, and at post treatment they did not decrease anxiety, depression [20] or improve quality of life [21].

While previous research has synthesized the evidence on exercise interventions in improving physical health outcomes, or mental health interventions in improving mental health outcomes in CYP with heart conditions, no review so far has evaluated the effectiveness of mental and physical health interventions for CYP with heart conditions.

As such, the aim of this review is to fill an important gap in the literature by addressing the following questions: (1) how effective are mental and physical health interventions in improving mental and physical health outcomes for young people with heart conditions? (2) do mental health interventions improve physical health outcomes and if so what is the size of the effect, (3) do exercise interventions improve mental health outcomes and if so what is the size of the effect.

Methods and analysis

This protocol is based on the Preferred Reporting Items for Systematic Reviews and Meta-Analysis Protocols (PRISMA-P) guidelines and review findings will be reported in line with PRISMA guidance [22]. The review has been registered with the International Prospective Register of Systematic Reviews (PROSPERO, registration number: CRD42022330582).

Search Strategy

The following databases will be searched: MEDLINE, EMBASE, PsycINFO, Web of Science, Cochrane Database of Systematic Reviews (via the Cochrane Library), Cochrane Central Register of

For peer review only

Controlled Trials (CENTRAL), DARE (via the Cochrane Library), and Science Citation Index (via Web of Science).

The search method will include extensive database searching and supplementary searching including forwards and backwards citation chasing, hand-searching of any key related reviews identified during the search process and additional searching on topic specific websites. The strategy will use both controlled headings (e.g., Medical Subject Headings (MeSH)) and free-text terms. Search terms will be grouped according to three concepts: heart conditions, mental health interventions, and exercise programs. Terms relating to these concepts will be developed based on the available literature. An example search strategy for PsychInfo can be seen in the supplemental file 1. The search will be adapted for each database.

Study Inclusion Criteria

Eligible studies will be peer-reviewed publications in English or Italian, that include young people (age 0-16 years old) diagnosed with any form of congenital heart disease (CHD) or an implanted cardiac device. Studies will be included if they are interventional studies designed to evaluate an exercise or physical activity intervention and/or a mental health intervention. Several interventional studies will be included such as: randomised controlled trials, benchmarking studies, non-randomised interventional studies, pilot studies, uncontrolled trials, and case series studies. To be included, studies must evaluate an exercise or physical activity intervention or psychosocial intervention. Exercise interventions may include a structured cardiac rehabilitation program. There are no restrictions on the setting of intervention, these can be home based, or centre based (i.e. hospital or community setting. A control group is not a necessary requirement for inclusion within this review. Comparator conditions may include standard care, waiting lists, active controls, no intervention control groups. Studies published before 1980 excluded as youth mental health not considered a discrete field prior to 1980 [23].

Information sources

For peer review only

An extensive database search will be conducted using MEDLINE, EMBASE, PsycINFO, Web of Science, Cochrane Database of Systematic Reviews (via the Cochrane Library), Cochrane Central Register of Controlled Trials (CENTRAL), DARE (via the Cochrane Library), and Science Citation Index (via Web of Science). Databases will be searched from inception to May 2022. The citation lists of included references will be checked and forwards citation searching (identifying where included references have been cited) will be carried out using Web of Science and Google Scholar. For the current review, any included articles will be subject to forward and backwards citation searching. Study authors will be contacted where information is missing and/or the full text article is unavailable.

Data management

The searches will be recorded using PRISMA guidelines, including the list of databases searched, recording of the dates (original and updated) searched and the strategies used for each database. All the articles identified by the database searches will be imported into EndNote reference management software X20 prior to de-duplication and screening. Duplicates not identified by Endnote will be manually removed. References identified by the updated search will be handled in the same manner. Articles will then be exported to Rayyan, a web-based software for the management of initial screening and selection of papers in systematic reviews [24].

Study selection

Article titles and abstracts will be screened against the inclusion/exclusion criteria by two independent reviewers (JA, AB). Following this, full texts of the remaining articles will be screened against the inclusion/exclusion criteria. Any conflicts or uncertainty will be resolved by discussion with the research team. All articles deemed to be included in the review will be discussed with the research team and any discrepancies of opinion will be resolved by LC and AW. Reasons for inclusion/exclusion will be recorded and a PRISMA flow chart will be used to show the details of the selection process.

For peer review only

Data extraction

One reviewer will independently extract data in each eligible study using a data extraction form. Other reviewers will check the extracted data to ensure accuracy and completeness of the data.

The following information will be extracted where available:

- General: Author, publication date, country of origin, DOI.
- Study Characteristics: Aims/Hypotheses, Study design, Inclusion/exclusion criteria, recruitment methods.
- Sample Characteristics: size, gender distribution, age, co-morbidity information and baseline clinical characteristics including cardiac diagnoses.
- Intervention: duration (number and length of sessions) mode of delivery, drop-out rates, attendance statistics, follow-up times (if applicable), quality of intervention using TIDieR checklist [25].
- Data analysis: comparison conditions (if applicable), statistical procedures used to analyse data.
- Primary and secondary outcomes.
- Key findings: pre- and post-treatment descriptive data, follow-up data, effect size of intervention.

Where a study is described across multiple publications, an attempt will be made to extract and combine all the available data. Study authors will be contacted if data is missing. If multiple papers are published using the same dataset only the study most relevant to the review will be included to avoid duplication of participants.

Outcomes

The most commonly reported outcomes for physical health are cardiorespiratory fitness (CRF) assessed by peak oxygen consumption and daily physical activity (subjective and objective outcomes). Primary outcomes for mental health are often quality of life, but we will consider available evidence on specific anxiety, depression, trauma and adjustment symptoms.

For peer review only

Additional outcomes are changes in health-related behaviours (physical activity, diet, smoking, alcohol, sedentary behaviour, and medication adherence) as interventions aimed at heart conditions would likely aim to improve modifiable CVD risk factors. Additional outcomes will include psychological well-being, lipid profile, blood pressure, weight/body mass index (BMI), number and type of cardiac events, rehospitalisation, and mortality.

Quality assessment/Risk of bias in individual studies

Quality of individual papers will be assessed using an appropriate Critical Appraisal tool based on study design as described in the manual for developing NICE guidelines[26], such as the Risk of Bias tool developed by The Cochrane Collaboration (version 2) [27]. One reviewer (JA) will complete the assessment and a second reviewer (BC) will check a random 20% of the studies. Intraclass correlation coefficient (ICC) will be used to indicate the level of agreement between the reviewer assessments, in a two-way random-effects model.

Data synthesis

A narrative review of the findings will be conducted. This will comprise information summarising study characteristics, patient characteristics, information pertaining to the delivery of exercise interventions (e.g., type of exercise, number of sessions, length of sessions, number of attendees, etc), outcome variables, follow-up, quality assessment, treatment effects (e.g., within- and between-group effect sizes). These will also be reported in tabular form.

If possible and appropriate, a random-effects model meta-analysis will be conducted to calculate the pooled within and between-group effect sizes. Between-group effect sizes will be calculated for the primary outcome measure in the treatment arm (exercise/mental health intervention) compared with waitlist control or active treatment control arms (at end of treatment and at longest follow-up time available) respectively; these will be analysed separately. Heterogeneity will be calculated using the I^2 statistic, where 25, 50, 75% are interpreted as referring to low, moderate, and high levels of heterogeneity (Higgins et al., 2019). Discussions of suitability of

For peer review only

inclusion of studies in any meta-analysis will be held with the research team. The meta-analysis will be conducted using Review Manager 5 (RevMan), according to the Cochrane guidelines [28].

For continuous variables, the mean difference will be calculated if the same measurement scale was used, alternatively the standardised mean difference (SMD) will be calculated (with 95% CI [confidence interval]). For dichotomous variables, proportions will be compared using risk ratios (with 95% CIs). If a meta-analysis is not possible, data will be summarised using a narrative synthesis approach.

Subgroup analyses will be conducted where appropriate and possible. This might consist of subgroups of populations (e.g., type of CHD, age), or intervention characteristics (home vs centre-based, aerobic vs resistance exercise, presence or not of psychological treatment elements)

Meta-bias(es)

Where there are a minimum of 10 studies available which allow for any between-group effect size calculations, publication bias will be investigated using visual inspection of funnel plots (looking for asymmetry indicative of publication bias).

Confidence in cumulative evidence

If appropriate and possible, the Grading of Recommendations Assessment, Development, and Evaluation guidelines (GRADE) approach will be used to make a judgement about the overall quality of the evidence base.

Patient and public involvement

Patients and the public will not be involved directly in the design and conduct of the review. However, the development of our review question was informed by focus groups held with CYP with heart conditions. The results of these interviews highlighted misconceptions regarding physical activity and exercise and pointed to unhelpful coping styles that could have significant psychosocial impact.

Ethics and Dissemination

For peer review only

Ethical approval is not required for this systematic review because primary data will not be collected. This systematic review protocol is registered in the International Prospective Register of Systematic Reviews (http://www.crd.york.ac.uk/PROSPERO). The result of the review will be disseminated through publication in an academic journal and disseminated as part of future work in the development of exercise-based interventions for CYP with CHD.

Discussion

Interventions that include physical and mental health support for CYP with heart conditions have the potential to inform future research and improve patient outcomes. The findings could contribute to overcoming barriers and misconceptions associated with exercise and physical activity thereby promoting healthier lifestyles in CYP with heart conditions.

The review will identify exercise and physical activity interventions, and mental-health interventions that are associated with change in important health outcomes in CYP with heart conditions. The results will provide key insights into the content and design of successful interventions for this patient group and help to inform the design of future exercise-based and integrative psychosocial interventions for CYP with CHD.

This review addresses a gap in the literature by evaluating effectiveness of exercise and mental-health interventions in this young patient group on both mental and physical health outcomes. This systematic review is being conducted in the broader context of developing a cardiac rehabilitation programme that includes physical and mental health support for CYP with heart conditions.

Data availability

No data are associated with this article.

Author Contributions:

LC and AW conceived the study, are co-chief investigators and wrote the initial grant protocol. All authors contributed to the first and subsequent drafts of the manuscript and approved the final

For peer review only

version for submission. JA contributed to the protocol methods, first and subsequent drafts of the protocol. BC and AB contributed to editing the manuscript.

Competing interests

The authors declare that they have no known competing financial interests or personal relationships that could have appeared to influence the work reported in this protocol.

Funding Statement: This study is funded by the National Institute for Health Research (grant number NIHR203634). The views expressed are those of the author(s) and not necessarily those of the NIHR or the Department of Health and Social Care.

For peer review only

References

[1] NCD Child, "Children & Non-Communicable Disease: Global Burden Report," 2019. Accessed: May 12, 2022. [Online]. Available: https://www.ncdchild.org/wp-content/uploads/2021/03/ncdchild_global_burden-report-2019.pdf

[2] D. S. Cooper *et al.*, "Pulmonary complications associated with the treatment of patients with congenital cardiac disease: consensus definitions from the Multi-Societal Database Committee for Pediatric and Congenital Heart Disease," *Cardiology in the Young*, vol. 18, no. S2, pp. 215–221, 2008, doi: 10.1017/S1047951108002941.

[3] M. Kamphuis, T. Vogels, J. Ottenkamp, E. E. van der Wall, S. P. Verloove-Vanhorick, and H. W. Vliegen, "Employment in Adults With Congenital Heart Disease," *Archives of Pediatrics & Adolescent Medicine*, vol. 156, no. 11, pp. 1143–1148, Nov. 2002, doi: 10.1001/ARCHPEDI.156.11.1143.

[4] D. Brandhagen, R. Feldt, and D. Williams, "Long-Term Psychologic Implications of Congenital Heart Disease: A 25-Year Follow-Up," *Mayo Clinic Proceedings*, vol. 66, no. 5, pp. 474–479, May 1991, doi: 10.1016/S0025-6196(12)62387-8.

[5] B. W. McCrindle *et al.*, "Physical activity levels in children and adolescents are reduced after the Fontan procedure, independent of exercise capacity, and are associated with lower perceived general health," *Archives of Disease in Childhood*, vol. 92, no. 6, pp. 509–514, Jun. 2007, doi: 10.1136/ADC.2006.105239.

[6] D. H. J. Thijssen, A. J. Maiorana, G. O'Driscoll, N. T. Cable, M. T. E. Hopman, and D. J. Green, "Impact of inactivity and exercise on the vasculature in humans," *European Journal of Applied Physiology*, vol. 108, no. 5, pp. 845–875, Mar. 2010, doi: 10.1007/S00421-009-1260-X/TABLES/1.

[7] T. Takken *et al.*, "Recommendations for physical activity, recreation sport, and exercise training in paediatric patients with congenital heart disease: a report from the Exercise, Basic & Translational Research Section of the European Association of Cardiovascular Prevention and Rehabilitation, the European Congenital Heart and Lung Exercise Group, and the Association for European Paediatric Cardiology," *European Journal of Preventive Cardiology*, vol. 19, no. 5, pp. 1034–1065, Oct. 2012, doi: 10.1177/1741826711420000.

[8] P. E. Longmuir *et al.*, "Promotion of Physical Activity for Children and Adults With Congenital Heart Disease," *Circulation*, vol. 127, no. 21, pp. 2147–2159, May 2013, doi: 10.1161/CIR.0B013E318293688F.

[9] M. Vigl, E. Niggemeyer, A. Hager, G. Schwedler, S. Kropf, and U. Bauer, "The importance of socio-demographic factors for the quality of life of adults with congenital heart disease," *Quality of Life Research*, vol. 20, no. 2, pp. 169–177, Mar. 2011, doi: 10.1007/S11136-010-9741-2/TABLES/5.

[10] B. Latal, S. Helfricht, J. E. Fischer, U. Bauersfeld, and M. A. Landolt, "Psychological adjustment and quality of life in children and adolescents following open-heart surgery for congenital heart disease: a systematic review," *BMC Pediatr*, vol. 9, no. 1, Jan. 2009, doi: 10.1186/1471-2431-9-6.

[11] P. Moons, K. van Deyk, K. Marquet, L. de Bleser, S. de Geest, and W. Budts, "Profile of adults with congenital heart disease having a good, moderate, or poor quality of life: a cluster

For peer review only

analytic study," *European journal of cardiovascular nursing*, vol. 8, no. 2, pp. 151–157, Jun. 2009, doi: 10.1016/J.EJCNURSE.2008.11.001.

[12] Q. Wang, M. Hay, D. Clarke, and S. Menahem, "The Prevalence and Predictors of Anxiety and Depression in Adolescents with Heart Disease," *The Journal of Pediatrics*, vol. 161, no. 5, pp. 943-946.e1, Nov. 2012, doi: 10.1016/J.JPEDS.2012.04.010.

[13] A. Abda, M. E. Bolduc, A. Tsimicalis, J. Rennick, D. Vatcher, and M. Brossard-Racine, "Psychosocial Outcomes of Children and Adolescents With Severe Congenital Heart Defect: A Systematic Review and Meta-Analysis," *J Pediatr Psychol*, vol. 44, no. 4, pp. 463–477, May 2019, doi: 10.1093/JPEPSY/JSY085.

[14] M. I. Awaad and K. E. Darahim, "Depression and anxiety in adolescents with congenital heart disease", doi: 10.1097/01.XME.0000457325.90630.4f.

[15] J. R. Moon, J. Huh, I. S. Kang, S. W. Park, T. G. Jun, and H. J. Lee, "Factors influencing depression in adolescents with congenital heart disease," *Heart & Lung*, vol. 38, no. 5, pp. 419–426, Sep. 2009, doi: 10.1016/J.HRTLNG.2008.11.005.

[16] A. W. van Deutekom and A. J. Lewandowski, "Physical activity modification in youth with congenital heart disease: a comprehensive narrative review," *Pediatr Res*, vol. 89, no. 7, pp. 1650–1658, May 2021, doi: 10.1038/S41390-020-01194-8.

[17] N. Duppen *et al.*, "Systematic review of the effects of physical exercise training programmes in children and young adults with congenital heart disease," *International Journal of Cardiology*, vol. 168, no. 3, pp. 1779–1787, Oct. 2013, doi: 10.1016/J.IJCARD.2013.05.086.

[18] K. Dulfer, W. A. Helbing, N. Duppen, and E. M. W. J. Utens, "Associations between exercise capacity, physical activity, and psychosocial functioning in children with congenital heart disease: a systematic review," *European Journal of Preventive Cardiology*, vol. 21, no. 10, pp. 1200–1215, Oct. 2014, doi: 10.1177/2047487313494030.

[19] S. Tesson, P. N. Butow, G. F. Sholler, L. Sharpe, A. H. Kovacs, and N. A. Kasparian, "Psychological interventions for people affected by childhood-onset heart disease: A systematic review," *Health Psychology*, vol. 38, no. 2, pp. 151–161, Feb. 2019, doi: 10.1037/HEA0000704.

[20] V. A. Freedenberg, P. S. Hinds, and E. Friedmann, "Mindfulness-Based Stress Reduction and Group Support Decrease Stress in Adolescents with Cardiac Diagnoses: A Randomized Two-Group Study," *Pediatric Cardiology*, vol. 38, no. 7, pp. 1415–1425, Oct. 2017, doi: 10.1007/S00246-017-1679-5/FIGURES/3.

[21] S. Lee, J. Lee, and J. Y. Choi, "The effect of a resilience improvement program for adolescents with complex congenital heart disease," *European Journal of Cardiovascular Nursing*, vol. 16, no. 4, pp. 290–298, Apr. 2017, doi: 10.1177/1474515116659836.

[22] D. Moher *et al.*, "Preferred reporting items for systematic review and meta-analysis protocols (PRISMA-P) 2015 statement," *Systematic Reviews*, vol. 4, no. 1, pp. 148–160, 2015, doi: 10.1186/2046-4053-4-1.

[23] S. E. Hetrick, A. G. Parker, P. Callahan, and R. Purcell, "Evidence mapping: illustrating an emerging methodology to improve evidence-based practice in youth mental health," *Journal*

For peer review only

of Evaluation in Clinical Practice, vol. 16, no. 6, pp. 1025–1030, 2010, doi: https://doi.org/10.1111/j.1365-2753.2008.01112.x.

[24] H. and F. Z. and E. A. Ouzzani Mourad and Hammady, “Rayyan—a web and mobile app for systematic reviews,” *Systematic Reviews*, vol. 5, no. 1, p. 210, 2016, doi: 10.1186/s13643-016-0384-4.

[25] T. C. Hoffmann *et al.*, “Better reporting of interventions: template for intervention description and replication (TIDieR) checklist and guide,” *BMJ*, vol. 348, 2014, doi: 10.1136/bmj.g1687.

[26] National Institute for Health and Care Excellence, “Reviewing research evidence,” in *Developing NICE guidelines: the manual*, 2022.

[27] J. Higgins, J. Savović, M. Page, R. Elbers, and J. Sterne, “Assessing risk of bias in a randomized trial.,” in *Cochrane Handbook for Systematic Reviews of Interventions version 6.3 (updated February 2022)*, J. Higgins, J. Thomas, J. Chandler, M. Cumpston, T. Li, M. Page, and V. Welch, Eds. Cochrane, 2022.

[28] J. Higgins *et al.*, *Cochrane Handbook for Systematic Reviews of Interventions version 6.3 (updated February 2022)*. Cochrane, 2022.

For peer review only

Appendix 1

Search Strategy

1. "psycho education" or "psychosocial" or "psychosocial*" or "Psycho* adj intervent* or program* or strategy* or support or treat* or therap* or counsel* or manage*"

2. "quality of life"

3. "exercise" or "sports" or "aerobic exercise" or "physical activity" or "cardiac rehabilitation" or "training" or "physical exercise" or "recreation" or "aerobic training" or "aerobic intervention" or "exercise prescription" or "activity" or "exercise performance" or "exercise rehab*" or "exercise programme" or "aerobic exercise training" or "physical training" or "High-Intensity Interval Training" or "HIIT" or "rehab" or "strength" or "endurance" or "resistance"

4. “congenital heart disease” OR “congenital heart diseases” OR “congenital heart defect” OR “congenital heart defects” OR “heart disease” OR “cardiomyopathy” OR “arrythmia” OR “cardiac defect”

5. “children” OR “adolescent” OR “adolescents: OR “young adult” OR “paedeatric” OR “youth”

6. 1 OR 2 OR 3

7. 4 AND 5

8. 6 AND 7
LIMIT: 1980 – Current

only

BMJ Open

Effectiveness of physical and mental health interventions for young people with heart conditions: protocol for a systematic review and meta-analysis

Journal:	*BMJ Open*
Manuscript ID	bmjopen-2022-067342.R1
Article Type:	Protocol
Date Submitted by the Author:	13-Nov-2022
Complete List of Authors:	Capobianco, Lora; Greater Manchester Mental Health NHS Foundation Trust, Research and Innovation; The University of Manchester<br>Adewusi, Joy; Greater Manchester Mental Health NHS Foundation Trust, Research and Innovation<br>Cooper, Beth; Greater Manchester Mental Health NHS Foundation Trust, Research and Innovation<br>Belcher, Andrew; Greater Manchester Mental Health NHS Foundation Trust, Research and Innovation<br>Wells, Adrian; The University of Manchester, Department of Clinical Psychology
**Primary Subject Heading**:	Cardiovascular medicine
Secondary Subject Heading:	Mental health, Rehabilitation medicine
Keywords:	MENTAL HEALTH, CARDIOLOGY, REHABILITATION MEDICINE, Congenital heart disease < CARDIOLOGY, Child & adolescent psychiatry < PSYCHIATRY

SCHOLARONE™
Manuscripts

Effectiveness of physical and mental health interventions for young people with heart conditions: protocol for a systematic review and meta-analysis

Lora Capobianco [1,2]

Joy Adewusi [1]

Beth Cooper [1]

Andrew Belcher [1]

Adrian Wells [1,2]

[1] Research and Innovation, Greater Manchester Mental Health NHS Foundation Trust, Manchester, UK

[2] School of Psychological Sciences, Faculty of Biology, Medicine and Health, The University of Manchester

Corresponding Author: Lora Capobianco, lora.capobianco@gmmh.nhs.uk

Word count: 2862, Figures: 0; Tables: 0

For peer review only

For peer review only - http://bmjopen.bmj.com/site/about/guidelines.xhtml

Abstract

Introduction: Cardiovascular disease is amongst the most common of non-communicable diseases, affecting 13.9 million children and young people (CYP) globally. Survival rates for CYP with heart conditions are rising, however support for adjusting to life with a heart condition is lacking, as such it is unsurprising that 1 in 3 suffer from anxiety, depression, or adjustment disorder. The proposed review aims to identify and assess the effectiveness of physical and mental health interventions across physical and mental health outcomes in young people with cardiac conditions using narrative synthesis and meta-analysis if appropriate.

Methods and analysis: Embase, Medline, PubMed, PsycINFO, Cochrane Databases, Web of Science and reference lists of relevant publications will be searched from inception to June 2022 for articles published in English or Italian. Screening, data extraction, intervention coding and risk of bias will be performed by two independent reviewers using an extraction checklist. Intervention content and features will be identified and reported using the Template for Intervention Description and Replication (TIDieR) checklist. A narrative review of the included studies will be conducted. If possible and appropriate, a random-effects model meta-analysis will be conducted to calculate the pooled within-group and between-group effect sizes for the primary outcome measures. If sufficient data is available, a sub-group meta-analysis will investigate whether specific intervention types are associated with different levels of intervention effectiveness.

Ethics and Dissemination: This systematic review does not directly involve the use of human beings therefore there is no requirement for ethical approval. Findings will be disseminated through peer-reviewed publication and in various media, such as conferences, congresses or symposia.

PROSPERO registration number: CRD42022330582

Keywords: cardiac, rehabilitation, CHD, mental health, adolescence

For peer review only

Strengths and limitations of this study:

- The review addresses a gap in the literature by being, to our knowledge the first to explore the effectiveness of mental and physical intervention on mental and physical outcomes in young people with a heart conditions and thereby overcoming a limitation of previous reviews.
- The review is not restricted to randomised trials but will incorporate a range of study designs including non-randomised interventional studies, pilot studies, uncontrolled trials, and randomised trials.
- While the study aims to evaluate the magnitude of the effect of current interventions, understanding of the mechanisms of change will not be evaluated but is an important area for future studies
- While the review aims to include a broad age range, young adults transitioning from paediatric to adult services have been excluded.

For peer review only

For peer review only - http://bmjopen.bmj.com/site/about/guidelines.xhtml

Introduction

Childhood heart conditions such as congenital heart disease, cardiomyopathy, and arrythmias, are amongst the most common childhood non communicable diseases, impacting 13.8 million children and young people (CYP) under the age of 20 globally [1]. Survivors of childhood heart conditions face challenges and comorbidities which impact their ability to function [2], future employment, and progression into independent adulthood [3], [4]. CYP with heart conditions are also less likely to engage in physical activity in comparison with healthy controls [5], which increases their risk for atherosclerosis, cardiovascular disease, obesity, and diabetes [6].

Recommendations from the American Heart Association and European Association of Cardiovascular Prevention and Rehabilitation [7] have noted the importance of CYP engaging in physical activity, exercise and maintaining a healthy lifestyle (i.e., healthy diet, engaging in exercise, reduced smoking and alcohol intake, and medication adherence). Regular moderate to vigorous physical activity in CYP has been shown to improve both physical and mental health [8]. This is of particular importance as CYP with heart conditions have a reduced quality of life [9]–[11] and poorer psychological functioning, with 30% suffering from anxiety or depression [10], [12]–[15] and 41% experience psychological maladjustment [10].

Interventions to improve outcomes in CYP with CHD have predominantly focused on improving exercise capacity or engagement in physical activity [16]. Van Deutekom and Lewandowski (2021) [16] conducted a narrative review of physical activity modification in CYP with CHD. Eight interventions were included that aimed to improve physical activity. The authors noted the heterogeneity in the interventions offered, with four of the trials reporting a significant increase in physical activity levels following the intervention. These results are in line with previous systematic reviews which have also noted that physical activity and exercise training programmes significantly improve physical health outcomes (i.e. peak VO_2, activity levels and muscle strength) [17].

For peer review only - http://bmjopen.bmj.com/site/about/guidelines.xhtml

While systematic reviews have noted that exercise and physical activity training improve physical health outcomes, only one review [18] has commented on improvements in quality-of-life, with one review conducted to evaluate mental health interventions for CYP with CHD [19]. Tesson *et al.* [19] conducted a systematic review of psychological interventions for childhood-onset heart disease and found only two studies focused on interventions for CYP [20], [21]. The results were disappointing, and at post treatment they did not decrease anxiety, depression [20] or improve quality of life [21].

Previous research has predominantly focused on synthesizing the evidence on exercise interventions in improving physical health outcomes, or mental health interventions in improving mental health outcomes in CYP with heart conditions. Reviews have not evaluated the effectiveness of such interventions and the size of the effect they produce. Interestingly, evidence from the wider literature (outside of cardiac health) suggests that exercise and physical activity interventions have produced significant improvements in anxiety and low mood [22-24]. Spruit et al [25] conducted a meta-analysis of 57 physical activity interventions and their effects on psychosocial outcomes including internalizing and externalizing problems. They found a small-to-medium effect size of physical activity interventions on reducing externalizing (Cohen's d = 0.320) and internalizing (Cohen's d = 0.316) problems in CYP. While the results are promising the effect sizes are limited, and are yet to outperform the benefits of mental health interventions on improving mental health outcomes. While these results are promising similar effects are yet to be explored in CYP with CHD. Furthermore, whilst physical activity/ exercise interventions appear to improve mental health outcomes, much less is known concerning the effect of mental health interventions on physical health outcomes.

As such, the aim of this review is to fill an important gap in the literature by addressing the following questions: (1) how effective are mental and physical health interventions in improving mental and physical health outcomes for young people with heart conditions? (2) do mental health

For peer review only - http://bmjopen.bmj.com/site/about/guidelines.xhtml

interventions improve physical health outcomes and if so what is the size of the effect, (3) do exercise interventions improve mental health outcomes and if so what is the size of the effect.

Methods and analysis

This protocol is based on the Preferred Reporting Items for Systematic Reviews and Meta-Analysis Protocols (PRISMA-P) guidelines (see Appendix 1) and review findings will be reported in line with PRISMA guidance [26]. The review has been registered with the International Prospective Register of Systematic Reviews (PROSPERO, registration number: CRD42022330582).

Search Strategy

The following databases will be searched: MEDLINE, EMBASE, PsycINFO, Web of Science, Cochrane Central Register of Controlled Trials (CENTRAL), DARE (via the Cochrane Library), and Science Citation Index (via Web of Science).

The search method will include extensive database searching and supplementary searching including forwards and backwards citation chasing, hand-searching of any key related reviews identified during the search process and additional searching on topic specific websites. The strategy will use both controlled headings (e.g., Medical Subject Headings (MeSH)) and free-text terms. Search terms will be grouped according to three concepts: heart conditions, mental health interventions, and exercise programs. Terms relating to these concepts will be developed based on the available literature. The search strategy can be seen in appendix 2.

Study Inclusion Criteria

Eligible studies will be peer-reviewed publications in English or Italian, that include young people (age 6-18 years old) diagnosed with any form of congenital heart disease (CHD) or an implanted cardiac device. Studies will be included if they are interventional studies designed to evaluate an exercise or physical activity intervention and/or a mental health intervention. Interventional studies will include: randomised controlled trials, non-randomised interventional

For peer review only - http://bmjopen.bmj.com/site/about/guidelines.xhtml

For peer review only

studies, pilot studies, and uncontrolled trials. To be included, studies must evaluate an exercise or physical activity intervention or psychosocial intervention. Exercise interventions may include a structured cardiac rehabilitation program. There are no restrictions on the setting of interventions, these can be home based, or centre based (i.e., hospital or community setting. A control group is not a necessary requirement for inclusion within this review. Comparator conditions may include standard care, waiting lists, active controls, no intervention control groups. Studies published before 1980 are excluded as youth mental health was not considered a discrete field prior to 1980 [27].

Information sources

An extensive database search will be conducted using MEDLINE, EMBASE, PsycINFO, Web of Science, Cochrane Database of Systematic Reviews (via the Cochrane Library), Cochrane Central Register of Controlled Trials (CENTRAL), DARE (via the Cochrane Library), and Science Citation Index (via Web of Science). Databases will be searched from inception to May 2022. The citation lists of included references will be checked and forwards citation searching (identifying where included references have been cited) will be carried out using Web of Science and Google Scholar. For the current review, any included articles will be subject to forward and backwards citation searching. Study authors will be contacted where information is missing and/or the full text article is unavailable.

Data management

The searches will be recorded using PRISMA guidelines, including the list of databases searched, recording of the dates (original and updated) searched and the strategies used for each database. All the articles identified by the database searches will be imported into EndNote reference management software X20 prior to de-duplication and screening. Duplicates not identified by Endnote will be manually removed. References identified by the updated search will be handled in the same manner. Articles will then be exported to Rayyan, a web-based software for the

For peer review only

For peer review only - http://bmjopen.bmj.com/site/about/guidelines.xhtml

management of initial screening and selection of papers in systematic reviews [28].Papers were then transferred to COVIDENCE to manage full-text review and data extraction [29].

Study selection

Article titles and abstracts will be screened against the inclusion/exclusion criteria by two independent reviewers (JA, AB). Following this, full texts of the remaining articles will be screened against the inclusion/exclusion criteria. Any conflicts or uncertainty will be resolved by discussion with the research team. All articles deemed to be included in the review will be discussed with the research team and any discrepancies of opinion will be resolved by LC and AW. Reasons for inclusion/exclusion will be recorded and a PRISMA flow chart will be used to show the details of the selection process.

Data extraction

One reviewer will independently extract data in each eligible study using a data extraction form. Other reviewers will check the extracted data to ensure accuracy and completeness of the data.

The following information will be extracted where available:

- General: Author, publication date, country of origin, DOI.
- Study Characteristics: Aims/Hypotheses, Study design, Inclusion/exclusion criteria, recruitment methods.
- Sample Characteristics: size, gender distribution, age, co-morbidity information and baseline clinical characteristics including cardiac diagnoses.
- Intervention: duration (number and length of sessions) mode of delivery, drop-out rates, attendance statistics, follow-up times (if applicable), quality of intervention using TIDieR checklist [25].
- Data analysis: comparison conditions (if applicable), statistical procedures used to analyse data.
- Primary and secondary outcomes.
- Key findings: pre- and post-treatment descriptive data, follow-up data, effect size of intervention.

For peer review only - http://bmjopen.bmj.com/site/about/guidelines.xhtml

Where a study is described across multiple publications, an attempt will be made to extract and combine all the available data. Study authors will be contacted if data is missing. If multiple papers are published using the same dataset only the study most relevant to the review will be included to avoid duplication of participants.

Outcomes

The most commonly reported outcomes for physical health are cardiorespiratory fitness (CRF) assessed by peak oxygen consumption and daily physical activity (subjective and objective outcomes). Primary outcomes for mental health are often quality of life, but we will consider available evidence on specific anxiety, depression, trauma and adjustment symptoms.

Additional outcomes are changes in health-related behaviours (physical activity, diet, smoking, alcohol, sedentary behaviour, and medication adherence) as interventions aimed at heart conditions would likely aim to improve modifiable CVD risk factors. Additional outcomes will include psychological well-being, lipid profile, blood pressure, weight/body mass index (BMI), number and type of cardiac events, rehospitalisation, and mortality.

Quality assessment/Risk of bias in individual studies

Quality of individual papers will be assessed using an appropriate Critical Appraisal tool based on study design as described in the manual for developing NICE guidelines[30], such as the Risk of Bias tool developed by The Cochrane Collaboration (version 2) [31]. One reviewer (JA) will complete the assessment and a second reviewer (BC) will check a random 20% of the studies. Intraclass correlation coefficient (ICC) will be used to indicate the level of agreement between the reviewer assessments, in a two-way random-effects model.

Data synthesis

A narrative review of the findings will be conducted. This will comprise information summarising study characteristics, patient characteristics, information pertaining to the delivery of exercise interventions (e.g., type of exercise, number of sessions, length of sessions, number of

For peer review only - http://bmjopen.bmj.com/site/about/guidelines.xhtml

attendees, etc), outcome variables, follow-up, quality assessment, treatment effects (e.g., within- and between-group effect sizes). These will also be reported in tabular form.

If possible and appropriate, a random-effects model meta-analysis will be conducted to calculate the pooled within and between-group effect sizes. Between-group effect sizes will be calculated for the primary outcome measure in the treatment arm (exercise/mental health intervention) compared with waitlist control or active treatment control arms (at end of treatment and at longest follow-up time available) respectively; these will be analysed separately. Heterogeneity will be calculated using the I^2 statistic, where 25, 50, 75% are interpreted as referring to low, moderate, and high levels of heterogeneity [32]. Discussions of suitability of inclusion of studies in any meta-analysis will be held with the research team. The meta-analysis will be conducted using Review Manager 5 (RevMan), according to the Cochrane guidelines [33].

For continuous variables, the mean difference will be calculated if the same measurement scale was used, alternatively the standardised mean difference (SMD) will be calculated (with 95% CI [confidence interval]). For dichotomous variables, proportions will be compared using risk ratios (with 95% CIs). If a meta-analysis is not possible, data will be summarised using a narrative synthesis approach.

Subgroup analyses will be conducted where appropriate and possible. This might consist of subgroups of populations (e.g., type of CHD, age), or intervention characteristics (home vs centre-based, aerobic vs resistance exercise, presence or not of psychological treatment elements)

Meta-bias(es)

Where there are a minimum of 10 studies available which allow for any between-group effect size calculations, publication bias will be investigated using visual inspection of funnel plots (looking for asymmetry indicative of publication bias).

Confidence in cumulative evidence

For peer review only - http://bmjopen.bmj.com/site/about/guidelines.xhtml

If appropriate and possible, the Grading of Recommendations Assessment, Development, and Evaluation guidelines (GRADE) approach will be used to make a judgement about the overall quality of the evidence base.

Patient and public involvement

Patients and the public will not be involved directly in the design and conduct of the review. However, the development of our review question was informed by a patient and public involvement group with CYP with heart conditions. The results of these interviews highlighted misconceptions regarding physical activity and exercise and pointed to unhelpful coping styles that could have significant psychosocial impact.

Ethics and Dissemination

Ethical approval is not required for this systematic review because primary data will not be collected. This systematic review protocol is registered in the International Prospective Register of Systematic Reviews (http://www.crd.york.ac.uk/PROSPERO). The result of the review will be disseminated through publication in an academic journal and disseminated as part of future work in the development of exercise-based interventions for CYP with CHD.

Discussion

Interventions that include physical and mental health support for CYP with heart conditions have the potential to inform future research and improve patient outcomes. The findings could contribute to overcoming barriers and misconceptions associated with exercise and physical activity thereby promoting healthier lifestyles in CYP with heart conditions.

The review will identify exercise and physical activity interventions, and mental-health interventions that are associated with change in important health outcomes in CYP with heart conditions. The results will provide key insights into the content and design of successful interventions for this patient group and help to inform the design of future exercise-based and integrative psychosocial interventions for CYP with CHD.

For peer review only - http://bmjopen.bmj.com/site/about/guidelines.xhtml

While the review aims to include a broad age range this may exclude young adults who are transitioning between paediatric and adult services and may exclude a population who may benefit from mental health and exercise interventions, as such future reviews should aim to evaluate those transitioning between services. In addition, while the aim of the review is to assess the nature and effectiveness of the interventions, less is likely to be known on the mechanisms of change that may lead to greater improvements in outcomes. As such, future reviews should aim to explore the mechanisms of change within interventions.

This review addresses a gap in the literature by evaluating effectiveness of exercise and mental-health interventions in this young patient group on both mental and physical health outcomes. This systematic review is being conducted in the broader context of developing a cardiac rehabilitation programme that includes physical and mental health support for CYP with heart conditions.

Author Contributions:

LC and AW conceived the study, are co-chief investigators and wrote the initial grant protocol. All authors contributed to the first and subsequent drafts of the manuscript and approved the final version for submission. JA contributed to the protocol methods, first and subsequent drafts of the protocol. BC and AB contributed to editing the manuscript.

Competing interests

The authors declare that they have no known competing financial interests or personal relationships that could have appeared to influence the work reported in this protocol.

Funding Statement: This study is funded by the National Institute for Health Research (grant number NIHR203634). The views expressed are those of the author(s) and not necessarily those of the NIHR or the Department of Health and Social Care.

For peer review only

For peer review only - http://bmjopen.bmj.com/site/about/guidelines.xhtml

References

[1] NCD Child, “Children & Non-Communicable Disease: Global Burden Report,” 2019. Accessed: May 12, 2022. [Online]. Available: https://www.ncdchild.org/wp-content/uploads/2021/03/ncdchild_global_burden-report-2019.pdf

[2] D. S. Cooper *et al.*, “Pulmonary complications associated with the treatment of patients with congenital cardiac disease: consensus definitions from the Multi-Societal Database Committee for Pediatric and Congenital Heart Disease,” *Cardiology in the Young*, vol. 18, no. S2, pp. 215–221, 2008, doi: 10.1017/S1047951108002941.

[3] M. Kamphuis, T. Vogels, J. Ottenkamp, E. E. van der Wall, S. P. Verloove-Vanhorick, and H. W. Vliegen, “Employment in Adults With Congenital Heart Disease,” *Archives of Pediatrics & Adolescent Medicine*, vol. 156, no. 11, pp. 1143–1148, Nov. 2002, doi: 10.1001/ARCHPEDI.156.11.1143.

[4] D. Brandhagen, R. Feldt, and D. Williams, “Long-Term Psychologic Implications of Congenital Heart Disease: A 25-Year Follow-Up,” *Mayo Clinic Proceedings*, vol. 66, no. 5, pp. 474–479, May 1991, doi: 10.1016/S0025-6196(12)62387-8.

[5] B. W. McCrindle *et al.*, “Physical activity levels in children and adolescents are reduced after the Fontan procedure, independent of exercise capacity, and are associated with lower perceived general health,” *Archives of Disease in Childhood*, vol. 92, no. 6, pp. 509–514, Jun. 2007, doi: 10.1136/ADC.2006.105239.

[6] D. H. J. Thijssen, A. J. Maiorana, G. O’Driscoll, N. T. Cable, M. T. E. Hopman, and D. J. Green, “Impact of inactivity and exercise on the vasculature in humans,” *European Journal of Applied Physiology*, vol. 108, no. 5, pp. 845–875, Mar. 2010, doi: 10.1007/S00421-009-1260-X/TABLES/1.

[7] T. Takken *et al.*, “Recommendations for physical activity, recreation sport, and exercise training in paediatric patients with congenital heart disease: a report from the Exercise, Basic & Translational Research Section of the European Association of Cardiovascular Prevention and Rehabilitation, the European Congenital Heart and Lung Exercise Group, and the Association for European Paediatric Cardiology,” *European Journal of Preventive Cardiology*, vol. 19, no. 5, pp. 1034–1065, Oct. 2012, doi: 10.1177/1741826711420000.

[8] P. E. Longmuir *et al.*, “Promotion of Physical Activity for Children and Adults With Congenital Heart Disease,” *Circulation*, vol. 127, no. 21, pp. 2147–2159, May 2013, doi: 10.1161/CIR.0B013E318293688F.

[9] M. Vigl, E. Niggemeyer, A. Hager, G. Schwedler, S. Kropf, and U. Bauer, “The importance of socio-demographic factors for the quality of life of adults with congenital heart disease,” *Quality of Life Research*, vol. 20, no. 2, pp. 169–177, Mar. 2011, doi: 10.1007/S11136-010-9741-2/TABLES/5.

[10] B. Latal, S. Helfricht, J. E. Fischer, U. Bauersfeld, and M. A. Landolt, “Psychological adjustment and quality of life in children and adolescents following open-heart surgery for congenital heart disease: a systematic review,” *BMC Pediatr*, vol. 9, no. 1, Jan. 2009, doi: 10.1186/1471-2431-9-6.

[11] P. Moons, K. van Deyk, K. Marquet, L. de Bleser, S. de Geest, and W. Budts, “Profile of adults with congenital heart disease having a good, moderate, or poor quality of life: a cluster

For peer review only

analytic study," *European journal of cardiovascular nursing*, vol. 8, no. 2, pp. 151–157, Jun. 2009, doi: 10.1016/J.EJCNURSE.2008.11.001.

[12] Q. Wang, M. Hay, D. Clarke, and S. Menahem, "The Prevalence and Predictors of Anxiety and Depression in Adolescents with Heart Disease," *The Journal of Pediatrics*, vol. 161, no. 5, pp. 943-946.e1, Nov. 2012, doi: 10.1016/J.JPEDS.2012.04.010.

[13] A. Abda, M. E. Bolduc, A. Tsimicalis, J. Rennick, D. Vatcher, and M. Brossard-Racine, "Psychosocial Outcomes of Children and Adolescents With Severe Congenital Heart Defect: A Systematic Review and Meta-Analysis," *J Pediatr Psychol*, vol. 44, no. 4, pp. 463–477, May 2019, doi: 10.1093/JPEPSY/JSY085.

[14] M. I. Awaad and K. E. Darahim, "Depression and anxiety in adolescents with congenital heart disease", doi: 10.1097/01.XME.0000457325.90630.4f.

[15] J. R. Moon, J. Huh, I. S. Kang, S. W. Park, T. G. Jun, and H. J. Lee, "Factors influencing depression in adolescents with congenital heart disease," *Heart & Lung*, vol. 38, no. 5, pp. 419–426, Sep. 2009, doi: 10.1016/J.HRTLNG.2008.11.005.

[16] A. W. van Deutekom and A. J. Lewandowski, "Physical activity modification in youth with congenital heart disease: a comprehensive narrative review," *Pediatr Res*, vol. 89, no. 7, pp. 1650–1658, May 2021, doi: 10.1038/S41390-020-01194-8.

[17] N. Duppen *et al.*, "Systematic review of the effects of physical exercise training programmes in children and young adults with congenital heart disease," *International Journal of Cardiology*, vol. 168, no. 3, pp. 1779–1787, Oct. 2013, doi: 10.1016/J.IJCARD.2013.05.086.

[18] K. Dulfer, W. A. Helbing, N. Duppen, and E. M. W. J. Utens, "Associations between exercise capacity, physical activity, and psychosocial functioning in children with congenital heart disease: a systematic review," *European Journal of Preventive Cardiology*, vol. 21, no. 10, pp. 1200–1215, Oct. 2014, doi: 10.1177/2047487313494030.

[19] S. Tesson, P. N. Butow, G. F. Sholler, L. Sharpe, A. H. Kovacs, and N. A. Kasparian, "Psychological interventions for people affected by childhood-onset heart disease: A systematic review," *Health Psychology*, vol. 38, no. 2, pp. 151–161, Feb. 2019, doi: 10.1037/HEA0000704.

[20] V. A. Freedenberg, P. S. Hinds, and E. Friedmann, "Mindfulness-Based Stress Reduction and Group Support Decrease Stress in Adolescents with Cardiac Diagnoses: A Randomized Two-Group Study," *Pediatric Cardiology*, vol. 38, no. 7, pp. 1415–1425, Oct. 2017, doi: 10.1007/S00246-017-1679-5/FIGURES/3.

[21] S. Lee, J. Lee, and J. Y. Choi, "The effect of a resilience improvement program for adolescents with complex congenital heart disease," *European Journal of Cardiovascular Nursing*, vol. 16, no. 4, pp. 290–298, Apr. 2017, doi: 10.1177/1474515116659836.

[22] Wassenaar TM, Williamson W, Johansen-Berg H, Dawes H, Roberts N, Foster C, Sexton CE. A critical evaluation of systematic reviews assessing the effect of chronic physical activity on academic achievement, cognition and the brain in children and adolescents: a systematic review. *International Journal of Behavio Nutrition & Physical Activity*, vol 17, no.1, pp79, Jun 2020, doi: 10.1186/s12966-020-00959-y.

For peer review only - http://bmjopen.bmj.com/site/about/guidelines.xhtml

[23] Andermo, S., Hallgren, M., Nguyen, TTD. *et al*. School-related physical activity interventions and mental health among children: a systematic review and meta-analysis. *Sports Medicine – Open, vol* 6, no. 25, Jun 2020, doi:10.1186/s40798-020-00254-x.

[24] Brown BM, Peiffer JJ, Sohrabi HR, Mondal A, Gupta VB, Rainey-Smith SR, Taddei K, Burnham S, Ellis KA, Szoeke C, Masters CL, Ames D, Rowe CC, Martins RN; AIBL research group. Intense physical activity is associated with cognitive performance in the elderly. *Translational Psychiatry, vol 2, no. 11, e191, Nov* 2012, doi: 10.1038/tp.2012.118.

[25] Spruit MA, Burtin C, De Boever P, Langer D, Vogiatzis I, Wouters EF, Franssen FM. COPD and exercise: does it make a difference? *Breathe (Sheff*), vol. 12, no. 2, e38-49, Jun 2016, doi:10.1183/20734735.003916.

[26] D. Moher *et al.*, "Preferred reporting items for systematic review and meta-analysis protocols (PRISMA-P) 2015 statement," *Systematic Reviews*, vol. 4, no. 1, pp. 148–160, 2015, doi: 10.1186/2046-4053-4-1.

[27] S. E. Hetrick, A. G. Parker, P. Callahan, and R. Purcell, "Evidence mapping: illustrating an emerging methodology to improve evidence-based practice in youth mental health," *Journal of Evaluation in Clinical Practice*, vol. 16, no. 6, pp. 1025–1030, 2010, doi: https://doi.org/10.1111/j.1365-2753.2008.01112.x.

[28] H. and F. Z. and E. A. Ouzzani Mourad and Hammady, "Rayyan—a web and mobile app for systematic reviews," *Systematic Reviews*, vol. 5, no. 1, p. 210, 2016, doi: 10.1186/s13643-016-0384-4.

[29] Covidence systematic review software, Veritas Health Innovation, Melbourne, Australia. Available at www.covidence.org.

[30] T. C. Hoffmann *et al.*, "Better reporting of interventions: template for intervention description and replication (TIDieR) checklist and guide," *BMJ*, vol. 348, 2014, doi: 10.1136/bmj.g1687.

[30] National Institute for Health and Care Excellence, "Reviewing research evidence," in *Developing NICE guidelines: the manual*, 2022.

[31] J. Higgins, J. Savović, M. Page, R. Elbers, and J. Sterne, "Assessing risk of bias in a randomized trial.," in *Cochrane Handbook for Systematic Reviews of Interventions version 6.3 (updated February 2022)*, J. Higgins, J. Thomas, J. Chandler, M. Cumpston, T. Li, M. Page, and V. Welch, Eds. Cochrane, 2022.

[32] Higgins JPT, Thomas J, Chandler J, Cumpston M, Li T, Page MJ, Welch VA (editors). *Cochrane Handbook for Systematic Reviews of Interventions*. 2nd Edition. Chichester (UK): John Wiley & Sons, 2019.

[33] J. Higgins *et al.*, *Cochrane Handbook for Systematic Reviews of Interventions version 6.3 (updated February 2022)*. Cochrane, 2022.

For peer review only

For peer review only - http://bmjopen.bmj.com/site/about/guidelines.xhtml

Appendix 2

Search Strategy (PsychInfo)

1. "psycho education" or "psychosocial" or "psychosocial*" or "Psycho* adj intervent* or program* or strategy* or support or treat* or therap* or counsel* or manage*"

2. "quality of life"

3. "exercise" or "sports" or "aerobic exercise" or "physical activity" or "cardiac rehabilitation" or "training" or "physical exercise" or "recreation" or "aerobic training" or "aerobic intervention" or "exercise prescription" or "activity" or "exercise performance" or "exercise rehab*" or "exercise programme" or "aerobic exercise training" or "physical training" or "High-Intensity Interval Training" or "HIIT" or "rehab" or "strength" or "endurance" or "resistance"

4. “congenital heart disease” OR “congenital heart diseases” OR “congenital heart defect” OR “congenital heart defects” OR “heart disease” OR “cardiomyopathy” OR “arrythmia” OR “cardiac defect”

5. “children” OR “adolescent” OR “adolescents” OR “young adult” OR “paedeatric” OR “youth”

6. 1 OR 2 OR 3

7. 4 AND 5

8. 6 AND 7
LIMIT: 1980 – Current

only

Search Strategy (MEDLINE)

1. "psycho education".mp. [mp=title, book title, abstract, original title, name of substance word, subject heading word, floating sub-heading word, keyword heading word, organism supplementary concept word, protocol supplementary concept word, rare disease supplementary concept word, unique identifier, synonyms]
2. "psychosocial".ab,ti.
3. "psycho* intervent*".ab,ti.
4. "psycho* program*".ab,ti.
5. "psycho* strategy ".ab,ti.
6. "psycho* support ".ab,ti.
7. "psycho* treat* ".ab,ti.
8. "psycho* therap* ".ab,ti.
9. "psycho* counsel* ".ab,ti.
10. "psycho* manage* ".ab,ti.
11. "quality of life".ab,ti.
12. "exercise".ab,ti.
13. "sports".ab,ti.
14. "aerobic exercise".ab,ti.
15. "physical activity".ab,ti.
16. "cardiac rehabilitation".ab,ti.
17. "physical exercise".ab,ti.
18. "recreation".ab,ti.
19. "aerobic training".ab,ti.
20. "aerobic intervention".ab,ti.
21. "exercise prescription".ab,ti.
22. "exercise performance".ab,ti.
23. "exercise rehab* ".ab,ti.
24. "exercise programme".ab,ti.
25. "aerobic exercise training".ab,ti.
26. "physical training".ab,ti.
27. "high-intensity interval training".ab,ti.
28. "HIIT".ab,ti.

For peer review only

29. "rehab".ab,ti.

30. "strength".ab,ti.

31. "endurance".ab,ti.

32. "congenital heart disease".ab,ti.

33. "congenital heart diseases".ab,ti.

34. "congenital heart defects".ab,ti.

35. "congenital heart defect".ab,ti.

36. "heart disease".ab,ti.

37. "cardiomyopathy".ab,ti.

38. "arrythmia".ab,ti.

39. "cardiac defect".ab,ti.

40. "children".ab,ti.

41. "adolescen* ".ab,ti.

42. "young adult".ab,ti.

43. "paedeatric".ab,ti.

44. "youth".ab,ti.

45. 1 or 2 or 3 or 4 or 5 or 6 or 7 or 8 or 9 or 10

46. 12 or 13 or 14 or 15 or 16 or 17 or 18 or 19 or 20 or 21 or 22 or 23 or 24 or 25 or 26 or 27 or 28 or 29 or 30 or 31

47. 32 or 33 or 34 or 35 or 36 or 37 or 38 or 39

48. 40 or 41 or 42 or 43 or 44

49. 11 or 45 or 46

50. 47 and 48

51. 49 and 50

52. limit 51 to (yr="1980 -Current" and (english or italian))

53. limit 52 to ("all child (0 to 18 years)" or "newborn infant (birth to 1 month)" or "infant (1 to 23 months)" or "preschool child (2 to 5 years)" or "child (6 to 12 years)" or "adolescent (13 to 18 years)")

For peer review only

Search Strategy (EMBASE)

1. "psycho education".mp. [mp=title, abstract, heading word, table of contents, key concepts, original title, tests & measures, mesh word]
2. "psychosocial".ab,ti.
3. "psycho* intervent*".ab,ti.
4. "psycho* program*".ab,ti.
5. "psycho* strategy ".ab,ti.
6. "psycho* support ".ab,ti.
7. "psycho* treat* ".ab,ti.
8. "psycho* therap* ".ab,ti.
9. "psycho* counsel* ".ab,ti.
10. "psycho* manage* ".ab,ti.
11. "quality of life".ab,ti.
12. "exercise".ab,ti.
13. "sports".ab,ti.
14. "aerobic exercise".ab,ti.
15. "physical activity".ab,ti.
16. "cardiac rehabilitation".ab,ti.
17. "physical exercise".ab,ti.
18. "recreation".ab,ti.
19. "aerobic training".ab,ti.
20. "aerobic intervention".ab,ti.
21. "exercise prescription".ab,ti.
22. "exercise performance".ab,ti.
23. "exercise rehab* ".ab,ti.
24. "exercise programme".ab,ti.
25. "aerobic exercise training".ab,ti.
26. "physical training".ab,ti.
27. "high-intensity interval training".ab,ti.
28. "HIIT".ab,ti.
29. "rehab".ab,ti.
30. "strength".ab,ti.
31. "endurance".ab,ti.

For peer review only

32. "congenital heart disease".ab,ti.

33. "congenital heart diseases".ab,ti.

34. "congenital heart defect".ab,ti.

35. "congenital heart defects".ab,ti.

36. "cardiomyopathy".ab,ti.

37. "arrythmia".ab,ti.

38. "cardiac defect".ab,ti.

39. "children".ab,ti.

40. "adolescen* ".ab,ti.

41. "young adult".ab,ti.

42. "youth".ab,ti.

43. "paedeatric".ab,ti.

44. 1 or 2 or 3 or 4 or 5 or 6 or 7 or 8 or 9 or 10

45. 12 or 13 or 14 or 15 or 16 or 17 or 18 or 19 or 20 or 21 or 22 or 23 or 24 or 25 or 26 or 27 or 28 or 29 or 30 or 31

46. 32 or 33 or 34 or 35 or 36 or 37 or 38

47. 39 or 40 or 41 or 42 or 43

48. 11 or 44 or 45

49. 44 or 45

50. 46 and 47

51. 48 and 50

52. 49 and 50

53. limit 52 to (english language or italian)

54. limit 53 to (child <unspecified age> or preschool child <1 to 6 years> or school child <7 to 12 years> or adolescent <13 to 17 years>)

For peer review only

Search Strategy (Web of Science, including Science Citation Index)

1: TS= ("psycho education" or "psychosocial" or "psychosocial*" or "Psycho* adj intervent* or program* or strategy* or support or treat* or therap* or counsel* or manage*")
Results: 142860

2: TS=("quality of life")
Results: 493264

3: TS=("exercise" or "sports" or "aerobic exercise" or "physical activity" or "cardiac rehabilitation" or "exercise training" or "physical exercise" or "recreation" or "aerobic training" or "aerobic intervention" or "exercise prescription" or "exercise performance" or "exercise rehab*" or "exercise programme" or "aerobic exercise training" or "physical training" or "High-Intensity Interval Training" or "HIIT" or "rehab" or "strength" or "endurance")

4: TS=(“congenital heart disease” OR “congenital heart diseases” OR “congenital heart defect” OR “congenital heart defects” OR “heart disease” OR “cardiomyopathy” OR “arrythmia” OR “cardiac defect”)
Editions: WOS.SCI

5: TS=(“children” OR “adolescent” OR “adolescents" OR “young adult” OR “paedeatric” OR “youth”).
Editions: WOS.SCI

6: #1 OR #3 Editions: WOS.SCI

7: #4 AND #5 Editions: WOS.SCI

8: #6 AND #7 Editions: WOS.SCI

9: #6 AND #7 and 1921 or 1923 or 1927 or 1965 or 1969 or 1970 or 1972 or 1974 or 1975 or 1977 or 1979 (Exclude – Publication Years) Editions: WOS.SCI

10: #6 AND #7 and 1921 or 1923 or 1927 or 1965 or 1969 or 1970 or 1972 or 1974 or 1975 or 1977 or 1979 (Exclude – Publication Years) and English or Italian (Languages) Editions: WOS.SCI

For peer review only

Search Strategy (Cochrane Central Register of Controlled Trials (CENTRAL))

#1 ("psycho education" or "psychosocial" or "psychosocial*" or "Psycho* adj intervent* or program* or strategy* or support or treat* or therap* or counsel* or manage*"):ti,ab,kw (Word variations have been searched)

#2 quality of life

#3 ("exercise" or "sports" or "aerobic exercise" or "physical activity" or "cardiac rehabilitation" or "training" or "physical exercise" or "recreation" or "aerobic training" or "aerobic intervention" or "exercise prescription" or "activity" or "exercise performance" or "exercise rehab*" or "exercise programme" or "aerobic exercise training" or "physical training" or "High-Intensity Interval Training" or "HIIT" or "rehab" or "strength" or "endurance" or "resistance"):ti,ab,kw

#4 “congenital heart disease” OR “congenital heart diseases” OR “congenital heart defect” OR “congenital heart defects” OR “heart disease” OR “cardiomyopathy” OR “arrythmia” OR “cardiac defect”

#5 “children” OR adolescen* OR “young adult” OR “paedeatric” OR “youth”

#6 #1 OR #2 OR #3

#7 #4 and #5

#8 #6 and #7 with Publication Year from 1980 to 2022, in Trials

For peer review only

Search strategy - DARE (via the Cochrane Library)

#1	("psycho education" or "psychosocial" or "psychosocial*" or "Psycho* adj intervent* or program* or strategy* or support or treat* or therap* or counsel* or manage*"):ti,ab,kw (Word variations have been searched)
#2	quality of life
#3	("exercise" or "sports" or "aerobic exercise" or "physical activity" or "cardiac rehabilitation" or "training" or "physical exercise" or "recreation" or "aerobic training" or "aerobic intervention" or "exercise prescription" or "activity" or "exercise performance" or "exercise rehab*" or "exercise programme" or "aerobic exercise training" or "physical training" or "High-Intensity Interval Training" or "HIIT" or "rehab" or "strength" or "endurance" or "resistance"):ti,ab,kw
#4	“congenital heart disease” OR “congenital heart diseases” OR “congenital heart defect” OR “congenital heart defects” OR “heart disease” OR “cardiomyopathy” OR “arrythmia” OR “cardiac defect”
#5	“children” OR adolescen* OR “young adult” OR “paedeatric” OR “youth”
#6	#1 OR #2 OR #3
#7	#4 and #5
#8	#6 and #7 with Publication Year from 1980 to 2022, in Cochrane Reviews

For peer review only

PRISMA-P (Preferred Reporting Items for Systematic review and Meta-Analysis Protocols) 2015 checklist: recommended items to address in a systematic review protocol*

Section and topic	Item No	Checklist item	Page
**ADMINISTRATIVE INFORMATION**			
Title:			
Identification	1a	Identify the report as a protocol of a systematic review	
Update	1b	If the protocol is for an update of a previous systematic review, identify as such	1
Registration	2	If registered, provide the name of the registry (such as PROSPERO) and registration number	6
Authors:			
Contact	3a	Provide name, institutional affiliation, e-mail address of all protocol authors; provide physical mailing address of corresponding author	1
Contributions	3b	Describe contributions of protocol authors and identify the guarantor of the review	12
Amendments	4	If the protocol represents an amendment of a previously completed or published protocol, identify as such and list changes; otherwise, state plan for documenting important protocol amendments	
Support:			
Sources	5a	Indicate sources of financial or other support for the review	12
Sponsor	5b	Provide name for the review funder and/or sponsor	12
Role of sponsor or funder	5c	Describe roles of funder(s), sponsor(s), and/or institution(s), if any, in developing the protocol	12
**INTRODUCTION**			
Rationale	6	Describe the rationale for the review in the context of what is already known	4-5
Objectives	7	Provide an explicit statement of the question(s) the review will address with reference to participants, interventions, comparators, and outcomes (PICO)	5
**METHODS**			
Eligibility criteria	8	Specify the study characteristics (such as PICO, study design, setting, time frame) and report characteristics (such as years considered, language, publication status) to be used as criteria for eligibility for the review	6-7
Information sources	9	Describe all intended information sources (such as electronic databases, contact with study authors, trial registers or other grey literature sources) with planned dates of coverage	7
Search strategy	10	Present draft of search strategy to be used for at least one electronic database, including planned limits, such that it could be repeated	S1
Study records:			
Data	11a	Describe the mechanism(s) that will be used to manage records and data throughout the review	7

For peer review only

management			
Selection process	11b	State the process that will be used for selecting studies (such as two independent reviewers) through each phase of the review (that is, screening, eligibility and inclusion in meta-analysis)	8
Data collection process	11c	Describe planned method of extracting data from reports (such as piloting forms, done independently, in duplicate), any processes for obtaining and confirming data from investigators	8
Data items	12	List and define all variables for which data will be sought (such as PICO items, funding sources), any pre-planned data assumptions and simplifications	8
Outcomes and prioritization	13	List and define all outcomes for which data will be sought, including prioritization of main and additional outcomes, with rationale	8
Risk of bias in individual studies	14	Describe anticipated methods for assessing risk of bias of individual studies, including whether this will be done at the outcome or study level, or both; state how this information will be used in data synthesis	9
Data synthesis	15a	Describe criteria under which study data will be quantitatively synthesised	10
	15b	If data are appropriate for quantitative synthesis, describe planned summary measures, methods of handling data and methods of combining data from studies, including any planned exploration of consistency (such as I^2, Kendall's τ)	10
	15c	Describe any proposed additional analyses (such as sensitivity or subgroup analyses, meta-regression)	10
	15d	If quantitative synthesis is not appropriate, describe the type of summary planned	
Meta-bias(es)	16	Specify any planned assessment of meta-bias(es) (such as publication bias across studies, selective reporting within studies)	11
Confidence in cumulative evidence	17	Describe how the strength of the body of evidence will be assessed (such as GRADE)	11

*** It is strongly recommended that this checklist be read in conjunction with the PRISMA-P Explanation and Elaboration (cite when available) for important clarification on the items. Amendments to a review protocol should be tracked and dated. The copyright for PRISMA-P (including checklist) is held by the PRISMA-P Group and is distributed under a Creative Commons Attribution Licence 4.0.**

From: Shamseer L, Moher D, Clarke M, Ghersi D, Liberati A, Petticrew M, Shekelle P, Stewart L, PRISMA-P Group. Preferred reporting items for systematic review and meta-analysis protocols (PRISMA-P) 2015: elaboration and explanation. BMJ. 2015 Jan 2;349(jan02 1):g7647.

For peer review only

BMJ Open

Effectiveness of physical and mental health interventions for young people with heart conditions: protocol for a systematic review and meta-analysis

Journal:	*BMJ Open*
Manuscript ID	bmjopen-2022-067342.R2
Article Type:	Protocol
Date Submitted by the Author:	09-Jan-2023

Complete List of Authors:	Capobianco, Lora; Greater Manchester Mental Health NHS Foundation Trust, Research and Innovation; The University of Manchester<br>Adewusi, Joy; Greater Manchester Mental Health NHS Foundation Trust, Research and Innovation<br>Cooper, Beth; Greater Manchester Mental Health NHS Foundation Trust, Research and Innovation<br>Belcher, Andrew; Greater Manchester Mental Health NHS Foundation Trust, Research and Innovation<br>Wells, Adrian; The University of Manchester, Department of Clinical Psychology

**Primary Subject Heading**:	Cardiovascular medicine
Secondary Subject Heading:	Mental health, Rehabilitation medicine
Keywords:	MENTAL HEALTH, CARDIOLOGY, REHABILITATION MEDICINE, Congenital heart disease < CARDIOLOGY, Child & adolescent psychiatry < PSYCHIATRY

SCHOLARONE™ Manuscripts

Effectiveness of physical and mental health interventions for young people with heart conditions: protocol for a systematic review and meta-analysis

Lora Capobianco [1,2]

Joy Adewusi [1]

Beth Cooper [1]

Andrew Belcher [1]

Adrian Wells [1,2]

[1] Research and Innovation, Greater Manchester Mental Health NHS Foundation Trust, Manchester, UK

[2] School of Psychological Sciences, Faculty of Biology, Medicine and Health, The University of Manchester

Correspondence to:

Lora Capobianco

lora.capobianco@gmmh.nhs.uk

Word count: 2862, Figures: 0; Tables: 0

Abstract

Introduction: Cardiovascular disease is amongst the most common of non-communicable diseases, affecting 13.9 million children and young people (CYP) globally. Survival rates for CYP with heart conditions are rising, however support for adjusting to life with a heart condition is lacking, as such it is unsurprising that 1 in 3 suffer from anxiety, depression, or adjustment disorder. The proposed review aims to identify and assess the effectiveness of physical and mental health interventions across physical and mental health outcomes in young people with cardiac conditions using narrative synthesis and meta-analysis if appropriate.

Methods and analysis: Embase, Medline, PubMed, PsycINFO, Cochrane Databases, Web of Science and reference lists of relevant publications will be searched from inception to June 2022 for articles published in English or Italian. Screening, data extraction, intervention coding and risk of bias will be

For peer review only - http://bmjopen.bmj.com/site/about/guidelines.xhtml

performed by two independent reviewers using an extraction checklist. Intervention content and features will be identified and reported using the Template for Intervention Description and Replication (TIDieR) checklist. A narrative review of the included studies will be conducted. If possible and appropriate, a random-effects model meta-analysis will be conducted to calculate the pooled within-group and between-group effect sizes for the primary outcome measures. If sufficient data is available, a sub-group meta-analysis will investigate whether specific intervention types are associated with different levels of intervention effectiveness.

Ethics and dissemination: This systematic review does not directly involve the use of human beings therefore there is no requirement for ethical approval. Findings will be disseminated through peer-reviewed publication and in various media, such as conferences, congresses or symposia.

PROSPERO registration number: CRD42022330582.

Keywords: cardiac, rehabilitation, CHD, mental health, adolescence

Strengths and limitations of this study:

- The review addresses a gap in the literature by being, to our knowledge, the first to explore the effectiveness of mental and physical intervention on mental and physical outcomes in young people with heart conditions, thereby overcoming a limitation of previous reviews.
- The review is not restricted to randomised trials but will incorporate a range of study designs including non-randomised interventional studies, pilot studies, uncontrolled trials, and randomised trials.
- While the study aims to evaluate the magnitude of the effect of current interventions, understanding of the mechanisms of change will not be evaluated but is an important area for future studies
- While the review aims to include a broad age range, studies of young adults transitioning from paediatric to adult services will be excluded.

For peer review only
For peer review only - http://bmjopen.bmj.com/site/about/guidelines.xhtml

For peer review only

For peer review only - http://bmjopen.bmj.com/site/about/guidelines.xhtml

INTRODUCTION

Childhood heart conditions such as congenital heart disease, cardiomyopathy, and arrythmias, are amongst the most common childhood non communicable diseases, impacting 13.8 million children and young people (CYP) under the age of 20 globally [1]. Survivors of childhood heart conditions face challenges and comorbidities which impact their ability to function [2], future employment, and progression into independent adulthood [3-4]. CYP with heart conditions are also less likely to engage in physical activity in comparison with healthy controls [5], which increases their risk for atherosclerosis, cardiovascular disease, obesity, and diabetes [6].

Recommendations from the American Heart Association and European Association of Cardiovascular Prevention and Rehabilitation [7] have noted the importance of CYP engaging in physical activity, exercise and maintaining a healthy lifestyle (i.e., healthy diet, engaging in exercise, reduced smoking and alcohol intake, and medication adherence). Regular moderate to vigorous physical activity in CYP has been shown to improve both physical and mental health [8]. This is of particular importance as CYP with heart conditions have a reduced quality of life [9-11] and poorer psychological functioning, with 30% suffering from anxiety or depression [10], [12-15] and 41% experience psychological maladjustment [10].

Interventions to improve outcomes in CYP with CHD have predominantly focused on improving exercise capacity or engagement in physical activity [16]. Van Deutekom and Lewandowski [16] conducted a narrative review of physical activity modification in CYP with CHD. Eight interventions were included that aimed to improve physical activity. The authors noted the heterogeneity in the interventions offered, with four of the trials reporting a significant increase in physical activity levels following the intervention. These results are in line with previous systematic reviews which have also noted that physical activity and exercise training programmes significantly improve physical health outcomes (i.e. peak VO_2, activity levels and muscle strength) [17].

For peer review only - http://bmjopen.bmj.com/site/about/guidelines.xhtml

While systematic reviews have noted that exercise and physical activity training improve physical health outcomes, only one review [18] has commented on improvements in quality-of-life, with one review conducted to evaluate mental health interventions for CYP with CHD [19]. Tesson *et al.* [19] conducted a systematic review of psychological interventions for childhood-onset heart disease and found only two studies focused on interventions for CYP [20], [21]. The results were disappointing, and at post treatment they did not decrease anxiety, depression [20] or improve quality of life [21].

Previous research has predominantly focused on synthesizing the evidence on exercise interventions in improving physical health outcomes, or mental health interventions in improving mental health outcomes in CYP with heart conditions. Reviews have not evaluated the effectiveness of such interventions and the size of the effect they produce. Interestingly, evidence from the wider literature (outside of cardiac health) suggests that exercise and physical activity interventions have produced significant improvements in anxiety and low mood [22-24]. Spruit et al [25] conducted a meta-analysis of 57 physical activity interventions and their effects on psychosocial outcomes including internalizing and externalizing problems. They found a small-to-medium effect size of physical activity interventions on reducing externalizing (Cohen's d = 0.320) and internalizing (Cohen's d = 0.316) problems in CYP. While the results are promising the effect sizes are limited and are yet to outperform the benefits of mental health interventions on improving mental health outcomes. While these results are promising similar effects are yet to be explored in CYP with CHD. Furthermore, whilst physical activity/ exercise interventions appear to improve mental health outcomes, much less is known concerning the effect of mental health interventions on physical health outcomes.

As such, the aim of this review is to fill an important gap in the literature by addressing the following questions: (1) how effective are mental and physical health interventions in improving mental and physical health outcomes for young people with heart conditions? (2) do mental health

For peer review only - http://bmjopen.bmj.com/site/about/guidelines.xhtml

interventions improve physical health outcomes and if so, what is the size of the effect, (3) do exercise interventions improve mental health outcomes and if so what is the size of the effect.

METHODS AND ANALYSIS

This protocol is based on the Preferred Reporting Items for Systematic Reviews and Meta-Analysis Protocols (PRISMA-P) guidelines (see Appendix 1) and review findings will be reported in line with PRISMA guidance [26]. The review has been registered with the International Prospective Register of Systematic Reviews (PROSPERO, registration number: CRD42022330582).

Search strategy

The following databases will be searched: MEDLINE, EMBASE, PsycINFO, Web of Science, Cochrane Central Register of Controlled Trials (CENTRAL), DARE (via the Cochrane Library), and Science Citation Index (via Web of Science).

The search method will include extensive database searching and supplementary searching including forwards and backwards citation chasing, hand-searching of any key related reviews identified during the search process and additional searching on topic specific websites. The strategy will use both controlled headings (e.g., Medical Subject Headings (MeSH)) and free-text terms. Search terms will be grouped according to three concepts: heart conditions, mental health interventions, and exercise programs. Terms relating to these concepts will be developed based on the available literature. The search strategy can be seen in appendix 2.

Study inclusion criteria

Eligible studies will be peer-reviewed publications in English or Italian, that include young people (age 6-18 years old) diagnosed with any form of congenital heart disease (CHD) or an implanted cardiac device. Studies will be included if they are interventional studies designed to evaluate an exercise or physical activity intervention and/or a mental health intervention. Interventional studies will include randomised controlled trials, non-randomised interventional studies, pilot studies, and

For peer review only

uncontrolled trials. To be included, studies must evaluate an exercise or physical activity intervention or psychosocial intervention. Exercise interventions may include a structured cardiac rehabilitation program. There are no restrictions on the setting of interventions, these can be home based, or centre based (i.e., hospital or community setting. A control group is not a necessary requirement for inclusion within this review. Comparator conditions may include standard care, waiting lists, active controls, no intervention control groups. Studies published before 1980 are excluded as youth mental health was not considered a discrete field prior to 1980 [27].

Information sources

An extensive database search will be conducted using MEDLINE, EMBASE, PsycINFO, Web of Science, Cochrane Database of Systematic Reviews (via the Cochrane Library), Cochrane Central Register of Controlled Trials (CENTRAL), DARE (via the Cochrane Library), and Science Citation Index (via Web of Science). Databases will be searched from inception to May 2022. The citation lists of included references will be checked and forwards citation searching (identifying where included references have been cited) will be carried out using Web of Science and Google Scholar. For the current review, any included articles will be subject to forward and backwards citation searching. Study authors will be contacted where information is missing and/or the full text article is unavailable.

Data management

The searches will be recorded using PRISMA guidelines, including the list of databases searched, recording of the dates (original and updated) searched and the strategies used for each database. All the articles identified by the database searches will be imported into EndNote reference management software X20 prior to de-duplication and screening. Duplicates not identified by Endnote will be manually removed. References identified by the updated search will be handled in the same manner. Articles will then be exported to Rayyan, a web-based software for the management of initial screening and selection of papers in systematic reviews [28].Papers will then be transferred to COVIDENCE to manage full-text review and data extraction [29].

For peer review only

Study selection

Article titles and abstracts will be screened against the inclusion/exclusion criteria by two independent reviewers (JA, AB). Following this, full texts of the remaining articles will be screened against the inclusion/exclusion criteria. Any conflicts or uncertainty will be resolved by discussion with the research team. All articles deemed to be included in the review will be discussed with the research team and any discrepancies of opinion will be resolved by LC and AW. Reasons for inclusion/exclusion will be recorded and a PRISMA flow chart will be used to show the details of the selection process.

Data extraction

One reviewer will independently extract data in each eligible study using a data extraction form. Other reviewers will check the extracted data to ensure accuracy and completeness of the data.

The following information will be extracted where available:

- General: Author, publication date, country of origin, DOI.
- Study Characteristics: Aims/Hypotheses, Study design, Inclusion/exclusion criteria, recruitment methods.
- Sample Characteristics: size, gender distribution, age, co-morbidity information and baseline clinical characteristics including cardiac diagnoses.
- Intervention: duration (number and length of sessions) mode of delivery, drop-out rates, attendance statistics, follow-up times (if applicable), quality of intervention using TIDieR checklist [25].
- Data analysis: comparison conditions (if applicable), statistical procedures used to analyse data.
- Primary and secondary outcomes.
- Key findings: pre- and post-treatment descriptive data, follow-up data, effect size of intervention.

Where a study is described across multiple publications, an attempt will be made to extract and combine all the available data. Study authors will be contacted if data is missing. If multiple papers

For peer review only - http://bmjopen.bmj.com/site/about/guidelines.xhtml

are published using the same dataset only the study most relevant to the review will be included to avoid duplication of participants.

Outcomes

The most commonly reported outcomes for physical health are cardiorespiratory fitness (CRF) assessed by peak oxygen consumption and daily physical activity (subjective and objective outcomes). Primary outcomes for mental health are often quality of life, but we will consider available evidence on specific anxiety, depression, trauma and adjustment symptoms.

Additional outcomes are changes in health-related behaviours (physical activity, diet, smoking, alcohol, sedentary behaviour, and medication adherence) as interventions aimed at heart conditions would likely aim to improve modifiable CVD risk factors. Additional outcomes will include psychological well-being, lipid profile, blood pressure, weight/body mass index (BMI), number and type of cardiac events, rehospitalisation, and mortality.

Quality assessment/risk of bias in individual studies

Quality of individual papers will be assessed using an appropriate Critical Appraisal tool based on study design as described in the manual for developing NICE guidelines[30], such as the Risk of Bias tool developed by The Cochrane Collaboration (version 2) [31]. One reviewer (JA) will complete the assessment and a second reviewer (BC) will check a random 20% of the studies. Intraclass correlation coefficient (ICC) will be used to indicate the level of agreement between the reviewer assessments, in a two-way random-effects model.

Data synthesis

A narrative review of the findings will be conducted. This will comprise information summarising study characteristics, patient characteristics, information pertaining to the delivery of exercise interventions (e.g., type of exercise, number of sessions, length of sessions, number of attendees, etc), outcome variables, follow-up, quality assessment, treatment effects (e.g., within-and between-group effect sizes). These will also be reported in tabular form.

For peer review only - http://bmjopen.bmj.com/site/about/guidelines.xhtml

If possible and appropriate, a random-effects model meta-analysis will be conducted to calculate the pooled within and between-group effect sizes. Between-group effect sizes will be calculated for the primary outcome measure in the treatment arm (exercise/mental health intervention) compared with waitlist control or active treatment control arms (at end of treatment and at longest follow-up time available) respectively; these will be analysed separately. Heterogeneity will be calculated using the I^2 statistic, where 25, 50, 75% are interpreted as referring to low, moderate, and high levels of heterogeneity [32]. Discussions of suitability of inclusion of studies in any meta-analysis will be held with the research team. The meta-analysis will be conducted using Review Manager 5 (RevMan), according to the Cochrane guidelines [33].

For continuous variables, the mean difference will be calculated if the same measurement scale was used, alternatively the standardised mean difference (SMD) will be calculated (with 95% CI [confidence interval]). For dichotomous variables, proportions will be compared using risk ratios (with 95% CIs). If a meta-analysis is not possible, data will be summarised using a narrative synthesis approach.

Subgroup analyses will be conducted where appropriate and possible. This might consist of subgroups of populations (e.g., type of CHD, age), or intervention characteristics (home vs centre-based, aerobic vs resistance exercise, presence or not of psychological treatment elements)

Meta-bias(es)

Where there are a minimum of 10 studies available which allow for any between-group effect size calculations, publication bias will be investigated using visual inspection of funnel plots (looking for asymmetry indicative of publication bias).

Confidence in cumulative evidence

If appropriate and possible, the Grading of Recommendations Assessment, Development, and Evaluation guidelines (GRADE) approach will be used to make a judgement about the overall quality of the evidence base.

For peer review only - http://bmjopen.bmj.com/site/about/guidelines.xhtml

Patient and public involvement

Patients and the public will not be involved directly in the design and conduct of the review. However, the development of our review question was informed by a patient and public involvement group with CYP with heart conditions. The results of these interviews highlighted misconceptions regarding physical activity and exercise and pointed to unhelpful coping styles that could have significant psychosocial impact.

ETHICS AND DISSEMINATION

Ethical approval is not required for this systematic review because primary data will not be collected. This systematic review protocol is registered in the International Prospective Register of Systematic Reviews (http://www.crd.york.ac.uk/PROSPERO). The result of the review will be disseminated through publication in an academic journal and disseminated as part of future work in the development of exercise-based interventions for CYP with CHD.

DISCUSSION

Interventions that include physical and mental health support for CYP with heart conditions have the potential to inform future research and improve patient outcomes. The findings could contribute to overcoming barriers and misconceptions associated with exercise and physical activity thereby promoting healthier lifestyles in CYP with heart conditions.

The review will identify exercise and physical activity interventions, and mental-health interventions that are associated with change in important health outcomes in CYP with heart conditions. The results will provide key insights into the content and design of successful interventions for this patient group and help to inform the design of future exercise-based and integrative psychosocial interventions for CYP with CHD.

While the review aims to include a broad age range this may exclude young adults who are transitioning between paediatric and adult services and may exclude a population who may benefit from mental health and exercise interventions, as such future reviews should aim to evaluate those

For peer review only - http://bmjopen.bmj.com/site/about/guidelines.xhtml

transitioning between services. In addition, while the aim of the review is to assess the nature and effectiveness of the interventions, less is likely to be known on the mechanisms of change that may lead to greater improvements in outcomes. As such, future reviews should aim to explore the mechanisms of change within interventions.

This review addresses a gap in the literature by evaluating effectiveness of exercise and mental-health interventions in this young patient group on both mental and physical health outcomes. This systematic review is being conducted in the broader context of developing a cardiac rehabilitation programme that includes physical and mental health support for CYP with heart conditions.

Contributors

LC and AW conceived the study, are co-chief investigators and wrote the initial grant protocol. All authors contributed to the first and subsequent drafts of the manuscript and approved the final version for submission. JA contributed to the protocol methods, first and subsequent drafts of the protocol. BC and AB contributed to editing the manuscript.

Competing interests

The authors declare that they have no known competing financial interests or personal relationships that could have appeared to influence the work reported in this protocol.

Funding

This study is funded by the National Institute for Health Research (grant number NIHR203634). The views expressed are those of the author(s) and not necessarily those of the NIHR or the Department of Health and Social Care.

For peer review only

References

[1] NCD Child, "Children & Non-Communicable Disease: Global Burden Report," 2019. Accessed: May 12, 2022. [Online]. Available: https://www.ncdchild.org/wp-content/uploads/2021/03/ncdchild_global_burden-report-2019.pdf

[2] D. S. Cooper *et al.*, "Pulmonary complications associated with the treatment of patients with congenital cardiac disease: consensus definitions from the Multi-Societal Database Committee for Pediatric and Congenital Heart Disease," *Cardiology in the Young*, vol. 18, no. S2, pp. 215–221, 2008, doi: 10.1017/S1047951108002941.

[3] M. Kamphuis, T. Vogels, J. Ottenkamp, E. E. van der Wall, S. P. Verloove-Vanhorick, and H. W. Vliegen, "Employment in Adults With Congenital Heart Disease," *Archives of Pediatrics & Adolescent Medicine*, vol. 156, no. 11, pp. 1143–1148, Nov. 2002, doi: 10.1001/ARCHPEDI.156.11.1143.

[4] D. Brandhagen, R. Feldt, and D. Williams, "Long-Term Psychologic Implications of Congenital Heart Disease: A 25-Year Follow-Up," *Mayo Clinic Proceedings*, vol. 66, no. 5, pp. 474–479, May 1991, doi: 10.1016/S0025-6196(12)62387-8.

[5] B. W. McCrindle *et al.*, "Physical activity levels in children and adolescents are reduced after the Fontan procedure, independent of exercise capacity, and are associated with lower perceived general health," *Archives of Disease in Childhood*, vol. 92, no. 6, pp. 509–514, Jun. 2007, doi: 10.1136/ADC.2006.105239.

[6] D. H. J. Thijssen, A. J. Maiorana, G. O'Driscoll, N. T. Cable, M. T. E. Hopman, and D. J. Green, "Impact of inactivity and exercise on the vasculature in humans," *European Journal of Applied Physiology*, vol. 108, no. 5, pp. 845–875, Mar. 2010, doi: 10.1007/S00421-009-1260-X/TABLES/1.

[7] T. Takken *et al.*, "Recommendations for physical activity, recreation sport, and exercise training in paediatric patients with congenital heart disease: a report from the Exercise, Basic & Translational Research Section of the European Association of Cardiovascular Prevention and Rehabilitation, the European Congenital Heart and Lung Exercise Group, and the Association for European Paediatric Cardiology," *European Journal of Preventive Cardiology*, vol. 19, no. 5, pp. 1034–1065, Oct. 2012, doi: 10.1177/1741826711420000.

[8] P. E. Longmuir *et al.*, "Promotion of Physical Activity for Children and Adults With Congenital Heart Disease," *Circulation*, vol. 127, no. 21, pp. 2147–2159, May 2013, doi: 10.1161/CIR.0B013E318293688F.

[9] M. Vigl, E. Niggemeyer, A. Hager, G. Schwedler, S. Kropf, and U. Bauer, "The importance of socio-demographic factors for the quality of life of adults with congenital heart disease," *Quality of Life Research*, vol. 20, no. 2, pp. 169–177, Mar. 2011, doi: 10.1007/S11136-010-9741-2/TABLES/5.

[10] B. Latal, S. Helfricht, J. E. Fischer, U. Bauersfeld, and M. A. Landolt, "Psychological adjustment and quality of life in children and adolescents following open-heart surgery for congenital heart disease: a systematic review," *BMC Pediatr*, vol. 9, no. 1, Jan. 2009, doi: 10.1186/1471-2431-9-6.

[11] P. Moons, K. van Deyk, K. Marquet, L. de Bleser, S. de Geest, and W. Budts, "Profile of adults with congenital heart disease having a good, moderate, or poor quality of life: a cluster

For peer review only - http://bmjopen.bmj.com/site/about/guidelines.xhtml

analytic study," *European journal of cardiovascular nursing*, vol. 8, no. 2, pp. 151–157, Jun. 2009, doi: 10.1016/J.EJCNURSE.2008.11.001.

[12] Q. Wang, M. Hay, D. Clarke, and S. Menahem, "The Prevalence and Predictors of Anxiety and Depression in Adolescents with Heart Disease," *The Journal of Pediatrics*, vol. 161, no. 5, pp. 943-946.e1, Nov. 2012, doi: 10.1016/J.JPEDS.2012.04.010.

[13] A. Abda, M. E. Bolduc, A. Tsimicalis, J. Rennick, D. Vatcher, and M. Brossard-Racine, "Psychosocial Outcomes of Children and Adolescents With Severe Congenital Heart Defect: A Systematic Review and Meta-Analysis," *J Pediatr Psychol*, vol. 44, no. 4, pp. 463–477, May 2019, doi: 10.1093/JPEPSY/JSY085.

[14] M. I. Awaad and K. E. Darahim, "Depression and anxiety in adolescents with congenital heart disease", doi: 10.1097/01.XME.0000457325.90630.4f.

[15] J. R. Moon, J. Huh, I. S. Kang, S. W. Park, T. G. Jun, and H. J. Lee, "Factors influencing depression in adolescents with congenital heart disease," *Heart & Lung*, vol. 38, no. 5, pp. 419–426, Sep. 2009, doi: 10.1016/J.HRTLNG.2008.11.005.

[16] A. W. van Deutekom and A. J. Lewandowski, "Physical activity modification in youth with congenital heart disease: a comprehensive narrative review," *Pediatr Res*, vol. 89, no. 7, pp. 1650–1658, May 2021, doi: 10.1038/S41390-020-01194-8.

[17] N. Duppen *et al.*, "Systematic review of the effects of physical exercise training programmes in children and young adults with congenital heart disease," *International Journal of Cardiology*, vol. 168, no. 3, pp. 1779–1787, Oct. 2013, doi: 10.1016/J.IJCARD.2013.05.086.

[18] K. Dulfer, W. A. Helbing, N. Duppen, and E. M. W. J. Utens, "Associations between exercise capacity, physical activity, and psychosocial functioning in children with congenital heart disease: a systematic review," *European Journal of Preventive Cardiology*, vol. 21, no. 10, pp. 1200–1215, Oct. 2014, doi: 10.1177/2047487313494030.

[19] S. Tesson, P. N. Butow, G. F. Sholler, L. Sharpe, A. H. Kovacs, and N. A. Kasparian, "Psychological interventions for people affected by childhood-onset heart disease: A systematic review," *Health Psychology*, vol. 38, no. 2, pp. 151–161, Feb. 2019, doi: 10.1037/HEA0000704.

[20] V. A. Freedenberg, P. S. Hinds, and E. Friedmann, "Mindfulness-Based Stress Reduction and Group Support Decrease Stress in Adolescents with Cardiac Diagnoses: A Randomized Two-Group Study," *Pediatric Cardiology*, vol. 38, no. 7, pp. 1415–1425, Oct. 2017, doi: 10.1007/S00246-017-1679-5/FIGURES/3.

[21] S. Lee, J. Lee, and J. Y. Choi, "The effect of a resilience improvement program for adolescents with complex congenital heart disease," *European Journal of Cardiovascular Nursing*, vol. 16, no. 4, pp. 290–298, Apr. 2017, doi: 10.1177/1474515116659836.

[22] Wassenaar TM, Williamson W, Johansen-Berg H, Dawes H, Roberts N, Foster C, Sexton CE. A critical evaluation of systematic reviews assessing the effect of chronic physical activity on academic achievement, cognition and the brain in children and adolescents: a systematic review. *International Journal of Behavio Nutrition & Physical Activity*, vol 17, no.1, pp79, Jun 2020, doi: 10.1186/s12966-020-00959-y.

For peer review only - http://bmjopen.bmj.com/site/about/guidelines.xhtml

[23] Andermo, S., Hallgren, M., Nguyen, TTD. *et al*. School-related physical activity interventions and mental health among children: a systematic review and meta-analysis. *Sports Medicine – Open, vol* 6, no. 25, Jun 2020, doi:10.1186/s40798-020-00254-x.

[24] Brown BM, Peiffer JJ, Sohrabi HR, Mondal A, Gupta VB, Rainey-Smith SR, Taddei K, Burnham S, Ellis KA, Szoeke C, Masters CL, Ames D, Rowe CC, Martins RN; AIBL research group. Intense physical activity is associated with cognitive performance in the elderly. *Translational Psychiatry, vol 2, no. 11, e191, Nov* 2012, doi: 10.1038/tp.2012.118.

[25] Spruit MA, Burtin C, De Boever P, Langer D, Vogiatzis I, Wouters EF, Franssen FM. COPD and exercise: does it make a difference? *Breathe (Sheff*), vol. 12, no. 2, e38-49, Jun 2016, doi:10.1183/20734735.003916.

[26] D. Moher *et al.*, "Preferred reporting items for systematic review and meta-analysis protocols (PRISMA-P) 2015 statement," *Systematic Reviews*, vol. 4, no. 1, pp. 148–160, 2015, doi: 10.1186/2046-4053-4-1.

[27] S. E. Hetrick, A. G. Parker, P. Callahan, and R. Purcell, "Evidence mapping: illustrating an emerging methodology to improve evidence-based practice in youth mental health," *Journal of Evaluation in Clinical Practice*, vol. 16, no. 6, pp. 1025–1030, 2010, doi: https://doi.org/10.1111/j.1365-2753.2008.01112.x.

[28] H. and F. Z. and E. A. Ouzzani Mourad and Hammady, "Rayyan—a web and mobile app for systematic reviews," *Systematic Reviews*, vol. 5, no. 1, p. 210, 2016, doi: 10.1186/s13643-016-0384-4.

[29] Covidence systematic review software, Veritas Health Innovation, Melbourne, Australia. Available at www.covidence.org.

[30] T. C. Hoffmann *et al.*, "Better reporting of interventions: template for intervention description and replication (TIDieR) checklist and guide," *BMJ*, vol. 348, 2014, doi: 10.1136/bmj.g1687.

[30] National Institute for Health and Care Excellence, "Reviewing research evidence," in *Developing NICE guidelines: the manual*, 2022.

[31] J. Higgins, J. Savović, M. Page, R. Elbers, and J. Sterne, "Assessing risk of bias in a randomized trial.," in *Cochrane Handbook for Systematic Reviews of Interventions version 6.3 (updated February 2022)*, J. Higgins, J. Thomas, J. Chandler, M. Cumpston, T. Li, M. Page, and V. Welch, Eds. Cochrane, 2022.

[32] Higgins JPT, Thomas J, Chandler J, Cumpston M, Li T, Page MJ, Welch VA (editors). *Cochrane Handbook for Systematic Reviews of Interventions*. 2nd Edition. Chichester (UK): John Wiley & Sons, 2019.

[33] J. Higgins *et al.*, *Cochrane Handbook for Systematic Reviews of Interventions version 6.3 (updated February 2022)*. Cochrane, 2022.

For peer review only - http://bmjopen.bmj.com/site/about/guidelines.xhtml

PRISMA-P (Preferred Reporting Items for Systematic review and Meta-Analysis Protocols) 2015 checklist: recommended items to address in a systematic review protocol*

Section and topic	Item No	Checklist item	Page
**ADMINISTRATIVE INFORMATION**			
Title:			
Identification	1a	Identify the report as a protocol of a systematic review	
Update	1b	If the protocol is for an update of a previous systematic review, identify as such	1
Registration	2	If registered, provide the name of the registry (such as PROSPERO) and registration number	6
Authors:			
Contact	3a	Provide name, institutional affiliation, e-mail address of all protocol authors; provide physical mailing address of corresponding author	1
Contributions	3b	Describe contributions of protocol authors and identify the guarantor of the review	12
Amendments	4	If the protocol represents an amendment of a previously completed or published protocol, identify as such and list changes; otherwise, state plan for documenting important protocol amendments	
Support:			
Sources	5a	Indicate sources of financial or other support for the review	12
Sponsor	5b	Provide name for the review funder and/or sponsor	12
Role of sponsor or funder	5c	Describe roles of funder(s), sponsor(s), and/or institution(s), if any, in developing the protocol	12
**INTRODUCTION**			
Rationale	6	Describe the rationale for the review in the context of what is already known	4-5
Objectives	7	Provide an explicit statement of the question(s) the review will address with reference to participants, interventions, comparators, and outcomes (PICO)	5
**METHODS**			
Eligibility criteria	8	Specify the study characteristics (such as PICO, study design, setting, time frame) and report characteristics (such as years considered, language, publication status) to be used as criteria for eligibility for the review	6-7
Information sources	9	Describe all intended information sources (such as electronic databases, contact with study authors, trial registers or other grey literature sources) with planned dates of coverage	7
Search strategy	10	Present draft of search strategy to be used for at least one electronic database, including planned limits, such that it could be repeated	S1
Study records:			
Data	11a	Describe the mechanism(s) that will be used to manage records and data throughout the review	7

For peer review only

management			
Selection process	11b	State the process that will be used for selecting studies (such as two independent reviewers) through each phase of the review (that is, screening, eligibility and inclusion in meta-analysis)	8
Data collection process	11c	Describe planned method of extracting data from reports (such as piloting forms, done independently, in duplicate), any processes for obtaining and confirming data from investigators	8
Data items	12	List and define all variables for which data will be sought (such as PICO items, funding sources), any pre-planned data assumptions and simplifications	8
Outcomes and prioritization	13	List and define all outcomes for which data will be sought, including prioritization of main and additional outcomes, with rationale	8
Risk of bias in individual studies	14	Describe anticipated methods for assessing risk of bias of individual studies, including whether this will be done at the outcome or study level, or both; state how this information will be used in data synthesis	9
Data synthesis	15a	Describe criteria under which study data will be quantitatively synthesised	10
	15b	If data are appropriate for quantitative synthesis, describe planned summary measures, methods of handling data and methods of combining data from studies, including any planned exploration of consistency (such as I^2, Kendall's τ)	10
	15c	Describe any proposed additional analyses (such as sensitivity or subgroup analyses, meta-regression)	10
	15d	If quantitative synthesis is not appropriate, describe the type of summary planned	
Meta-bias(es)	16	Specify any planned assessment of meta-bias(es) (such as publication bias across studies, selective reporting within studies)	11
Confidence in cumulative evidence	17	Describe how the strength of the body of evidence will be assessed (such as GRADE)	11

*** It is strongly recommended that this checklist be read in conjunction with the PRISMA-P Explanation and Elaboration (cite when available) for important clarification on the items. Amendments to a review protocol should be tracked and dated. The copyright for PRISMA-P (including checklist) is held by the PRISMA-P Group and is distributed under a Creative Commons Attribution Licence 4.0.**

From: Shamseer L, Moher D, Clarke M, Ghersi D, Liberati A, Petticrew M, Shekelle P, Stewart L, PRISMA-P Group. Preferred reporting items for systematic review and meta-analysis protocols (PRISMA-P) 2015: elaboration and explanation. BMJ. 2015 Jan 2;349(jan02 1):g7647.

For peer review only

Appendix 2

Search Strategy (PsychInfo)

1. "psycho education" or "psychosocial" or "psychosocial*" or "Psycho* adj intervent* or program* or strategy* or support or treat* or therap* or counsel* or manage*"

2. "quality of life"

3. "exercise" or "sports" or "aerobic exercise" or "physical activity" or "cardiac rehabilitation" or "training" or "physical exercise" or "recreation" or "aerobic training" or "aerobic intervention" or "exercise prescription" or "activity" or "exercise performance" or "exercise rehab*" or "exercise programme" or "aerobic exercise training" or "physical training" or "High-Intensity Interval Training" or "HIIT" or "rehab" or "strength" or "endurance" or "resistance"

4. “congenital heart disease” OR “congenital heart diseases” OR “congenital heart defect” OR “congenital heart defects” OR “heart disease” OR “cardiomyopathy” OR “arrythmia” OR “cardiac defect”

5. “children” OR “adolescent” OR “adolescents” OR “young adult” OR “paedeatric” OR “youth”

6. 1 OR 2 OR 3

7. 4 AND 5

8. 6 AND 7
LIMIT: 1980 – Current

only

Search Strategy (MEDLINE)

1. "psycho education".mp. [mp=title, book title, abstract, original title, name of substance word, subject heading word, floating sub-heading word, keyword heading word, organism supplementary concept word, protocol supplementary concept word, rare disease supplementary concept word, unique identifier, synonyms]
2. "psychosocial".ab,ti.
3. "psycho* intervent*".ab,ti.
4. "psycho* program*".ab,ti.
5. "psycho* strategy ".ab,ti.
6. "psycho* support ".ab,ti.
7. "psycho* treat* ".ab,ti.
8. "psycho* therap* ".ab,ti.
9. "psycho* counsel* ".ab,ti.
10. "psycho* manage* ".ab,ti.
11. "quality of life".ab,ti.
12. "exercise".ab,ti.
13. "sports".ab,ti.
14. "aerobic exercise".ab,ti.
15. "physical activity".ab,ti.
16. "cardiac rehabilitation".ab,ti.
17. "physical exercise".ab,ti.
18. "recreation".ab,ti.
19. "aerobic training".ab,ti.
20. "aerobic intervention".ab,ti.
21. "exercise prescription".ab,ti.
22. "exercise performance".ab,ti.
23. "exercise rehab* ".ab,ti.
24. "exercise programme".ab,ti.
25. "aerobic exercise training".ab,ti.
26. "physical training".ab,ti.
27. "high-intensity interval training".ab,ti.
28. "HIIT".ab,ti.

For peer review only

29. "rehab".ab,ti.

30. "strength".ab,ti.

31. "endurance".ab,ti.

32. "congenital heart disease".ab,ti.

33. "congenital heart diseases".ab,ti.

34. "congenital heart defects".ab,ti.

35. "congenital heart defect".ab,ti.

36. "heart disease".ab,ti.

37. "cardiomyopathy".ab,ti.

38. "arrythmia".ab,ti.

39. "cardiac defect".ab,ti.

40. "children".ab,ti.

41. "adolescen* ".ab,ti.

42. "young adult".ab,ti.

43. "paedeatric".ab,ti.

44. "youth".ab,ti.

45. 1 or 2 or 3 or 4 or 5 or 6 or 7 or 8 or 9 or 10

46. 12 or 13 or 14 or 15 or 16 or 17 or 18 or 19 or 20 or 21 or 22 or 23 or 24 or 25 or 26 or 27 or 28 or 29 or 30 or 31

47. 32 or 33 or 34 or 35 or 36 or 37 or 38 or 39

48. 40 or 41 or 42 or 43 or 44

49. 11 or 45 or 46

50. 47 and 48

51. 49 and 50

52. limit 51 to (yr="1980 -Current" and (english or italian))

53. limit 52 to ("all child (0 to 18 years)" or "newborn infant (birth to 1 month)" or "infant (1 to 23 months)" or "preschool child (2 to 5 years)" or "child (6 to 12 years)" or "adolescent (13 to 18 years)")

For peer review only

Search Strategy (EMBASE)

1. "psycho education".mp. [mp=title, abstract, heading word, table of contents, key concepts, original title, tests & measures, mesh word]
2. "psychosocial".ab,ti.
3. "psycho* intervent*".ab,ti.
4. "psycho* program*".ab,ti.
5. "psycho* strategy ".ab,ti.
6. "psycho* support ".ab,ti.
7. "psycho* treat* ".ab,ti.
8. "psycho* therap* ".ab,ti.
9. "psycho* counsel* ".ab,ti.
10. "psycho* manage* ".ab,ti.
11. "quality of life".ab,ti.
12. "exercise".ab,ti.
13. "sports".ab,ti.
14. "aerobic exercise".ab,ti.
15. "physical activity".ab,ti.
16. "cardiac rehabilitation".ab,ti.
17. "physical exercise".ab,ti.
18. "recreation".ab,ti.
19. "aerobic training".ab,ti.
20. "aerobic intervention".ab,ti.
21. "exercise prescription".ab,ti.
22. "exercise performance".ab,ti.
23. "exercise rehab* ".ab,ti.
24. "exercise programme".ab,ti.
25. "aerobic exercise training".ab,ti.
26. "physical training".ab,ti.
27. "high-intensity interval training".ab,ti.
28. "HIIT".ab,ti.
29. "rehab".ab,ti.
30. "strength".ab,ti.
31. "endurance".ab,ti.

For peer review only

32. "congenital heart disease".ab,ti.

33. "congenital heart diseases".ab,ti.

34. "congenital heart defect".ab,ti.

35. "congenital heart defects".ab,ti.

36. "cardiomyopathy".ab,ti.

37. "arrythmia".ab,ti.

38. "cardiac defect".ab,ti.

39. "children".ab,ti.

40. "adolescen* ".ab,ti.

41. "young adult".ab,ti.

42. "youth".ab,ti.

43. "paedeatric".ab,ti.

44. 1 or 2 or 3 or 4 or 5 or 6 or 7 or 8 or 9 or 10

45. 12 or 13 or 14 or 15 or 16 or 17 or 18 or 19 or 20 or 21 or 22 or 23 or 24 or 25 or 26 or 27 or 28 or 29 or 30 or 31

46. 32 or 33 or 34 or 35 or 36 or 37 or 38

47. 39 or 40 or 41 or 42 or 43

48. 11 or 44 or 45

49. 44 or 45

50. 46 and 47

51. 48 and 50

52. 49 and 50

53. limit 52 to (english language or italian)

54. limit 53 to (child <unspecified age> or preschool child <1 to 6 years> or school child <7 to 12 years> or adolescent <13 to 17 years>)

For peer review only

Search Strategy (Web of Science, including Science Citation Index)

1: TS= ("psycho education" or "psychosocial" or "psychosocial*" or "Psycho* adj intervent* or program* or strategy* or support or treat* or therap* or counsel* or manage*")
Results: 142860

2: TS=("quality of life")
Results: 493264

3: TS=("exercise" or "sports" or "aerobic exercise" or "physical activity" or "cardiac rehabilitation" or "exercise training" or "physical exercise" or "recreation" or "aerobic training" or "aerobic intervention" or "exercise prescription" or "exercise performance" or "exercise rehab*" or "exercise programme" or "aerobic exercise training" or "physical training" or "High-Intensity Interval Training" or "HIIT" or "rehab" or "strength" or "endurance")

4: TS=("congenital heart disease" OR "congenital heart diseases" OR "congenital heart defect" OR "congenital heart defects" OR "heart disease" OR "cardiomyopathy" OR "arrythmia" OR "cardiac defect")
Editions: WOS.SCI

5: TS=("children" OR "adolescent" OR "adolescents" OR "young adult" OR "paedeatric" OR "youth").
Editions: WOS.SCI

6: #1 OR #3 Editions: WOS.SCI

7: #4 AND #5 Editions: WOS.SCI

8: #6 AND #7 Editions: WOS.SCI

9: #6 AND #7 and 1921 or 1923 or 1927 or 1965 or 1969 or 1970 or 1972 or 1974 or 1975 or 1977 or 1979 (Exclude – Publication Years) Editions: WOS.SCI

10: #6 AND #7 and 1921 or 1923 or 1927 or 1965 or 1969 or 1970 or 1972 or 1974 or 1975 or 1977 or 1979 (Exclude – Publication Years) and English or Italian (Languages) Editions: WOS.SCI

For peer review only

Search Strategy (Cochrane Central Register of Controlled Trials (CENTRAL))

#1 ("psycho education" or "psychosocial" or "psychosocial*" or "Psycho* adj intervent* or program* or strategy* or support or treat* or therap* or counsel* or manage*"):ti,ab,kw (Word variations have been searched)

#2 quality of life

#3 ("exercise" or "sports" or "aerobic exercise" or "physical activity" or "cardiac rehabilitation" or "training" or "physical exercise" or "recreation" or "aerobic training" or "aerobic intervention" or "exercise prescription" or "activity" or "exercise performance" or "exercise rehab*" or "exercise programme" or "aerobic exercise training" or "physical training" or "High-Intensity Interval Training" or "HIIT" or "rehab" or "strength" or "endurance" or "resistance"):ti,ab,kw

#4 “congenital heart disease” OR “congenital heart diseases” OR “congenital heart defect” OR “congenital heart defects” OR “heart disease” OR “cardiomyopathy” OR “arrythmia” OR “cardiac defect”

#5 “children” OR adolescen* OR “young adult” OR “paedeatric” OR “youth”

#6 #1 OR #2 OR #3

#7 #4 and #5

#8 #6 and #7 with Publication Year from 1980 to 2022, in Trials

For peer review only

Search strategy - DARE (via the Cochrane Library)

#1 ("psycho education" or "psychosocial" or "psychosocial*" or "Psycho* adj intervent* or program* or strategy* or support or treat* or therap* or counsel* or manage*"):ti,ab,kw (Word variations have been searched)

#2 quality of life

#3 ("exercise" or "sports" or "aerobic exercise" or "physical activity" or "cardiac rehabilitation" or "training" or "physical exercise" or "recreation" or "aerobic training" or "aerobic intervention" or "exercise prescription" or "activity" or "exercise performance" or "exercise rehab*" or "exercise programme" or "aerobic exercise training" or "physical training" or "High-Intensity Interval Training" or "HIIT" or "rehab" or "strength" or "endurance" or "resistance"):ti,ab,kw

#4 “congenital heart disease” OR “congenital heart diseases” OR “congenital heart defect” OR “congenital heart defects” OR “heart disease” OR “cardiomyopathy” OR “arrythmia” OR “cardiac defect”

#5 “children” OR adolescen* OR “young adult” OR “paedeatric” OR “youth”

#6 #1 OR #2 OR #3

#7 #4 and #5

#8 #6 and #7 with Publication Year from 1980 to 2022, in Cochrane Reviews

For peer review only
